# Identification of human genetic variants modulating the course of COVID-19 infection with importance in other viral infections

**DOI:** 10.3389/fgene.2023.1240245

**Published:** 2023-08-29

**Authors:** Lana Salihefendić, Ivana Čeko, Larisa Bešić, Naida Mulahuseinović, Selma Durgut, Dino Pećar, Lejla Prnjavorac, Enis Kandić, Neven Meseldžić, Tamer Bego, Besim Prnjavorac, Damir Marjanović, Rijad Konjhodžić, Adna Ašić

**Affiliations:** ^1^ ALEA Genetic Center, Sarajevo, Bosnia and Herzegovina; ^2^ Department of Genetics and Bioengineering, International Burch University, Sarajevo, Bosnia and Herzegovina; ^3^ General Hospital Tešanj, Tešanj, Bosnia and Herzegovina; ^4^ Department of Pharmaceutical Biochemistry and Laboratory Diagnostics, Faculty of Pharmacy, University of Sarajevo, Sarajevo, Bosnia and Herzegovina; ^5^ Institute for Anthropological Research, University of Zagreb, Zagreb, Croatia

**Keywords:** *ACE2*, COVID-19, host genetics, *IRF7*, SARS-CoV-2, *TMPRSS2*

## Abstract

**Introduction:** COVID-19 has been a major focus of scientific research since early 2020. Due to its societal, economic, and clinical impact worldwide, research efforts aimed, among other questions, to address the effect of host genetics in susceptibility and severity of COVID-19.

**Methods:** We, therefore, performed next-generation sequencing of coding and regulatory regions of 16 human genes, involved in maintenance of the immune system or encoding receptors for viral entry into the host cells, in a subset of 60 COVID-19 patients from the General Hospital Tešanj, Bosnia and Herzegovina, classified into three groups of clinical conditions of different severity (“mild,” “moderate,” and “severe”).

**Results:** We confirmed that the male sex and older age are risk factors for severe clinical picture and identified 13 variants on seven genes (*CD55, IL1B, IL4, IRF7, DDX58, TMPRSS2*, and *ACE2*) with potential functional significance, either as genetic markers of modulated susceptibility to SARS-CoV-2 infection or modifiers of the infection severity. Our results include variants reported for the first time as potentially associated with COVID-19, but further research and larger patient cohorts are required to confirm their effect.

**Discussion:** Such studies, focused on candidate genes and/or variants, have a potential to answer the questions regarding the effect of human genetic makeup on the expected infection outcome. In addition, loci we identified here were previously reported to have clinical significance in other diseases and viral infections, thus confirming a general, broader significance of COVID-19-related research results following the end of the pandemic period.

## 1 Introduction

COVID-19, caused by the SARS-CoV-2 virus outbreak in Wuhan, China, was declared a public health emergency of international concern on 30 January 2020, and a pandemic on 12 March 2020, by the World Health Organization (WHO) (Huang et al., 2020). The genome of SARS-CoV-2, at 29,881 nt and 9,860 amino acids, is a larger linear single-stranded RNA viral genome (Cheng et al., 2020). It encodes four structural proteins (spike S, envelope E, nucleocapsid N, and membrane M) and sixteen non-structural proteins (labeled Nsp1-16) (Naqvi et al., 2020; Wang et al., 2020).

COVID-19 represents an unprecedented challenge to governments all around the world due to virus transmissibility, symptom variability and severity, uncertainty regarding the immunity development following the course of infection, and the overall impact on healthcare systems and global economy (Fauci et al., 2020). For that reason, multiple parallel scientific approaches were used to address the issue as rapidly and efficiently as possible. In this context, scientists around the world sequenced the viral genome. For example, the Global Initiative on Sharing Avian Influenza Data (GISAID) has over 15,700,000 sequenced SARS-CoV-2 viruses, as of July 2023, and the number of submissions is growing daily (Elbe and Buckland-Merrett, 2017). On the other hand, huge variability in the severity of clinical picture of COVID-19 has also been investigated. It was accepted that environmental, demographic, and clinical factors all have an impact on severity of COVID-19, but that the host genetics may also have a significant role in the severity, as well as susceptibility to SARS-CoV-2 infection at the first place (Docherty et al., 2020). It is well-stablished that more severe symptoms and higher mortality rate are both observed in older patients (over 60 years of age), males, and people with other comorbidities, such as diabetes mellitus, cardiovascular diseases, and respiratory diseases, among others (Wang et al., 2020).

Therefore, the aim of our research was to perform the first study of COVID-19 host genetics in Bosnia and Herzegovina, and the Western Balkans region, by sequencing the coding and regulatory regions of 16 human genes in COVID-19 patients classified into three groups of mild, moderate, and severe clinical picture of the disease, in order to establish whether any of detected genetic variants can be associated with severity of COVID-19 and/or susceptibility to the infection. Study genes are mostly involved in maintenance and homeostasis of human immune system or are producing viral (co-) receptors expressed on the surface of human cells.

## 2 Materials and methods

### 2.1 Ethical considerations and sample collection

Ethical approvals for conducting this study were granted by the Joint Ethics Committee of the General Hospital Tešanj, Bosnia and Herzegovina, for patient DNA sample and clinical record use (11 January 2021, document number 01-4-17/21) and the Ethics Committee of the Faculty of Engineering and Natural Sciences, International Burch University Sarajevo, Bosnia and Herzegovina, for conducting the molecular analyses (23 March 2021, document number 04-51/21). Prior to sample collection, all patients signed an informed consent form, while physicians in charge filled in the patient questionnaire regarding general demographic characteristics, clinical presentation of COVID-19, and comorbidities. This research was carried out in accordance with the Declaration of Helsinki.

Whole blood samples were collected from RT-PCR-confirmed COVID-19 patients (*n* = 60) in the General Hospital Tešanj from March to October 2021, stored at −20°C, and delivered on ice to the ALEA Genetic Center (Sarajevo) laboratory. Based on the patients’ symptoms, overall condition, oxygen saturation levels, and laboratory and radiological testing results, samples were classified into three groups: mild (*n* = 20), moderate (*n* = 20), and severe symptoms group (*n* = 20), based on Baj et al., 2020.

Following sample delivery to the DNA laboratory, they were de-frosted, and DNA was extracted immediately using the QIAamp DNA Mini Kit (Qiagen, Hilden, Germany). The original manufacturer’s protocol was modified only by using 100 µL of ATE buffer, instead of 200 μL, since DNA was not extracted right after sampling. Following extraction, DNA was quantified using Qubit™ 3.0 Fluorometer with dsDNA high-sensitivity (HS) kit (Thermo Fisher Scientific, Waltham, MA, United States).

### 2.2 Next-generation sequencing

Ion AmpliSeq Designer (Thermo Fisher Scientific) was used to create primers for 16 selected genes and their regulatory sequences, namely *HLA-A, HLA-B, HLA-C, ACE2, IL-6, IL-4, TMPRSS2, IFITM3, IL-12, DDX58, IRF-7, IRF-9, IL-1B, IL-1A, CD55*, and *TNF-α*. This custom-made primer panel was received frozen in two primer pools, whereby pool 1 contained 93 amplicons and pool 2 contained 92 amplicons. Candidate gene selection was performed in early 2020, meaning that we mainly relied on previously published literature regarding the effect of host genetics on SARS-CoV and MERS-CoV infections or the earliest reports related to biomarker changes that could potentially be indicative of variable immunological response to SARS-CoV-2 infection (Chapman and Hill, 2012; Trowsdale and Knight, 2013; Asselta et al., 2020; Ghafouri-Fard et al., 2020; Lingeswaran et al., 2020; Lipworth et al., 2020; Zhang et al., 2020; Zhou et al., 2020).

Library preparation was done using Ion AmpliSeq™ Library Kit 2.0 (Thermo Fisher Scientific) according to manufacturer’s instructions. The starting amount of DNA material was ranging from 30 to 100 ng, and the number of cycles was set to 24. Amplicon digestion, adapter ligation, and purification steps were performed according to manufacturer’s instructions. Product clean-up was done using Agencourt™ AMPure™ XP Reagent (Beckman Coulter, Brea, CA, United States). Following purification, libraries were quantified using real-time PCR and Ion Library TaqMan^®^ Quantitation Kit (Thermo Fisher Scientific) according to manufacturer’s instructions. Libraries with concentration over 100 pM were diluted to 100 pM and pooled together before emulsion PCR and enrichment, which was done using Ion Chef System (Thermo Fisher Scientific). The chip was loaded automatically with Ion Chef System using Ion 510™ and Ion 520™ and Ion 530™ Kit (Thermo Fisher Scientific). Next-generation sequencing (NGS) was performed using Ion GeneStudio™ S5 System and data was analyzed using Torrent Browser Software (Thermo Fisher Scientific) through VCF (Variant Caller Files) format and Coverage Analysis.

Further modifications were made on the library preparation protocol, as it was concluded that primer pool 2 had lower coverage (more information was published in Salihefendić et al., 2022). Data analysis was ultimately done on 48 samples containing sequences from both primer pools and 12 samples containing only pool 1 amplicons.

Finally, clinical exome analysis was performed on three samples from the severe clinical symptoms group, using TruSight One Sequencing Panel (Illumina, San Diego, CA, United States), according to manufacturer’s instructions. Sequencing of these samples was performed on Illumina MiSeq platform.

All sequences can be accessed within the Sequence Read Archive (SRA) repository on the National Center for Biotechnology information (NCBI) website, as detailed below.

### 2.3 Confirmatory Sanger sequencing

Genetic variants obtained through NGS were confirmed and the custom-made panel was validated via Sanger sequencing of five selected SNPs. Table 1 gives the position of these SNPs, their rs numbers, annealing temperatures, and designed primers.

**TABLE 1:** Designed primers for confirmatory Sanger sequencing and information about corresponding SNPs.

Gene	Position (GRCh37)	rs number	Nucleotide change	Primer pair (forward and reverse)	Annealing temperature (°C)
*TMPRSS2*	21:42845383	rs17854725	A>G	F 5′-GAC​CTG​CTC​AAG​GTC​ACA-3′	60
				R 5′-TGT​CCT​TGC​AGC​CTG​GG-3′	
*TMPRSS2*	21:42852497	rs12329760	C>T	F 5′-AGC​ACT​CAT​GTG​CCG​GTG-3′	58
				R 5′-TGG​ATA​ATC​CTC​CCT​CTG​G-3′	
*ACE2*	X:15610348	rs2285666	C>T	F 5′-CAT​TCA​TGT​CCT​TGC​CCT​TA-3′	56
				R 5′-GTG​GTC​AAA​AGG​ATA​TCT​TTA​T-3′	
*IFITM3*	11:320007	rs370862493	G>A	F 5′-GGA​TGC​CCA​GAA​TCA​GGG-3′	58
				R 5′-TGA​GGA​AGG​GGA​GGA​GGT-3′	
*IL4*	5:132018169	rs2243290	C>A	F 5′-GAC​AAG​TGC​CAC​AGT​AGG-3′	58
				R 5′-GCT​CGA​ACA​CTT​TGA​ATA​TTT-3′	

For every SNP, 10 samples were selected, taking care that both wild-type and variant allele-containing genotypes were selected. For genotypes with variant alleles, both heterozygous and homozygous individuals were selected, whenever possible. For PCR amplification, the final concentrations of 1x PCR Master Mix (Thermo Fisher Scientific) and 1 µM of both forward and reverse primers were used in a reaction of a total volume of 25 μL, including 10 ng of DNA. Initial denaturation was performed at 95°C for 3 min, followed by 40 cycles of denaturation at 95°C for 30 s, annealing for 30 s, and elongation at 72°C for 1 min. Final elongation was done at 72°C for 10 min.

Cycle sequencing was done using BigDye™ Terminator v3.1 Cycle Sequencing Kit (Thermo Fisher Scientific) according to manufacturer’s instructions. Products were purified using Macherey-Nagel™ NucleoSpin™ Gel and PCR Clean-up columns (Macherey-Nagel, Düren, Germany) and Sanger sequencing was performed on SeqStudio™ Genetic Analyzer (Thermo Fisher Scientific).

### 2.4 Statistical analysis

Statistical analysis was performed using the chi-square test of goodness-of-fit to compare clinical severity categories between male and female participants, as well as among the patients with different comorbidities (cardiovascular, metabolic, respiratory, and other comorbidities). The same test was used to investigate the influence of detected genetic variants on the severity of COVID-19 presentation and infection susceptibility. We have used the exact test of goodness-of-fit to perform age-, sex-, and comorbidity-stratified analyses to determine the effect of these variables on the differences in the frequency of appearance of genetic variants detected previously. One-way ANOVA was used to compare the mean age among the different clinical severity groups. Kruskal-Wallis test was deployed as a non-parametric alternative to one-way ANOVA, whereby it assumes that mean ranks are the same among the three age groups. In all analyses, *p*-value of 0.05 was considered critical for detection of significant differences between the study groups.

## 3 Results

In this study, we analyzed the effect of demographic characteristics, comorbidities, and genetic background of patients on severity of and susceptibility to COVID-19. The population age was ranging from 15 to 80, with median of 62.5, mean of 59.067, and standard deviation of 15.029, at a 95% confidence interval of 3.88 (upper 62.95, lower 55.18). Our results demonstrated that the male sex and older age are risk factors for severe symptoms of COVID-19 (Table 2).

**TABLE 2:** Demographic characteristics and comorbidities compared between three study groups. *p*-values denoting statistically significant differences between the study groups are bolded.

	Total (*n* = 60)	Mild symptoms (*n* = 20)	Moderate symptoms (*n* = 20)	Severe symptoms (n = 20)
Sex distribution				
**Male**	35 (58.33%)	11 (55.00%)	10 (50.00%)	14 (70.00%)
**Female**	25 (41.67%)	9 (45.00%)	10 (50.00%)	6 (30.00%)
*p*-value	0.096	0.317	1.000	**0.000063**
**Age distribution**				
**Mean age**	59.067	48.55	61.00	67.65
*p*-value		Mild vs. moderate	Moderate vs. severe	Mild vs. severe
		**0.010439**	**0.040**	**9.70 x 10** ^**−5**^
**Comorbidities statistics**				
**Cardiovascular**	32 (53.33%)	6 (30.00%)	11 (55.00%)	15 (75.00%)
*p*-value		Mild vs. moderate	Moderate vs. severe	Mild vs. severe
		**0.006695**	0.079	**0.000011**
**Respiratory**	11 (18.33%)	0 (0.00%)	4 (20.00%)	7 (35.00%)
*p*-value		Mild vs. moderate	Moderate vs. severe	Mild vs. severe
		**7.74 x 10** ^**−6**^	**0.043**	**3.30 x 10** ^**−9**^
**Metabolic**	17 (28.33%)	0 (0.00%)	9 (45.00%)	8 (40.00%)
*p*-value		Mild vs. moderate	Moderate vs. severe	Mild vs. severe
		**1.97 x 10** ^**−11**^	0.588	**2.54 x 10** ^**−10**^
**Other**	19 (31.67%)	4 (20.00%)	4 (20.00%)	11 (55.00%)
*p*-value		Mild vs. moderate	Moderate vs. severe	Mild vs. severe
		1.000	**0.000053**	**0.000053**

By collecting patients’ clinical records, we were able to analyze the frequency of observed comorbidities in three clinical groups. Cardiovascular comorbidities were significantly more common in severe when compared to mild and moderate symptom groups, and include hypertension, chronic cardiomyopathy, brain stroke, angina pectoris, atrial fibrillation, aortic aneurysm, and myocardial infarction. Respiratory comorbidities, including bronchitis, asthma, and history of tuberculosis, were more common in moderate and severe groups, when compared to mild, but also in the moderate symptom group when compared to severe. We have also detected significantly less patients with metabolic comorbidities in mild symptom group when compared to both moderate and severe groups. These comorbidities include diabetes, hypothyroidism, glucose intolerance and chronic sideropenic anemia (iron insufficiency). Other comorbidities encompass rheumatoid arthritis, renal insufficiency, acute liver lesion, chronic gastritis, and hepatitis C infection, and were assessed together. We found that these comorbidities were significantly more common in severe symptom group when compared to either mild or moderate groups (Table 2).

**FIGURE 1:**
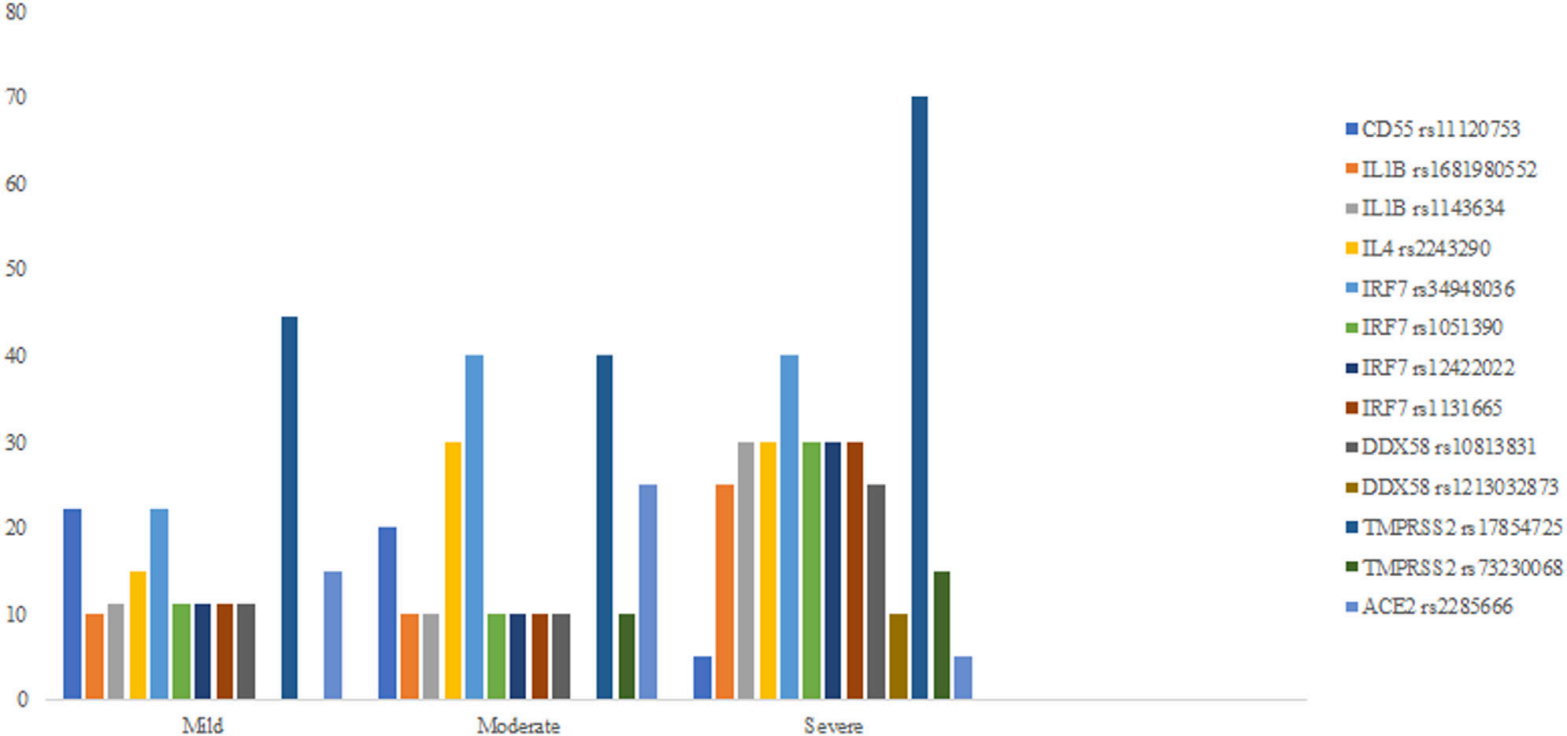
Percent fractions of detected genetic polymorphisms with significant differences in frequency between mild, moderate, and severe symptom groups.

In 11 of the study genes, we have observed genetic variants in our patients. Observed variants include single nucleotide polymorphisms, insertions, deletions, and complex variants (Table 3). We have detected variants that might be predisposing the patients towards milder or more severe symptoms of COVID-19, based on significant differences in the frequency of appearance of the study variants between the defined clinical groups. In our analyses, we grouped heterozygous and homozygous carriers of the variants together (Table 4; Figure 1). The full list of detected variants and accompanying statistics are given in Supplementary Table S1, while the results of clinical exome sequencing for three patients and observed allele frequencies of thus detected variants for European populations are given in Supplementary Table S2. We did not detect linkage disequilibrium or any other significant relationship between the viral-entry-associated genes (*ACE2* and *TMPRSS2*) and the remaining, immunity-related genes, in terms of mutation distribution in individual patients and within the cohort (data not shown).

**TABLE 3:** The number of patients and percentage of the total patient population in which variants on 13 study genes were observed, regardless of the genotype, and the total number of variants observed in the custom-made panel.

Gene	Number of patients with detected changes	Number of detected variants
*CD55*	23 (38.33%)	5
*IL1A*	35 (58.33%)	3
*IL1B*	26 (43.33%)	6
*IL2*	0 (0.00%)	0
*IL4*	21 (35.00%)	4
*IL6*	2 (3.33%)	2
*IRF7*	45 (75.00%)	16
*IRF9*	0 (0.00%)	0
*TNFα*	2 (3.33%)	1
*DDX58*	26 (43.33%)	18
*TMPRSS2*	58 (96.67%)	23
*ACE2*	13 (21.67%)	3
*IFITM3*	16 (26.67%)	5

**TABLE 4:** Detected genetic polymorphisms with significant differences in frequency of appearance between the study groups. Frequencies are given as percentage of all tested patients per study group in which variant was detected, regardless of genotype. *p*-values denoting statistically significant differences between the study groups are bolded.

rs number	Position	Change	Mild symptom group (%)	Moderate symptom group (%)	Severe symptom group (%)
*CD55*					
rs11120753	1:207527285	G>A	22.2	20	5
*p*-value			Mild vs. moderate	Moderate vs. severe	Mild vs. severe
			0.735	**0.0027**	**0.000974**
***IL1B***					
rs1681980552	2:113588756	delAAA	10	10	25
*p*-value			Mild vs. moderate	Moderate vs. severe	Mild vs. severe
			1.000	**0.011**	**0.011**
rs1143634	2:113,590,390	G>A	11.11	10	30
*p*-value			Mild vs. moderate	Moderate vs. severe	Mild vs. severe
			0.809	**0.001565**	**0.00322**
***IL4***					
rs2243290	5:132018169	C>A	15.00	30.00	30.00
*p*-value			Mild vs. moderate	Moderate vs. severe	Mild vs. severe
			**0.025**	1.000	**0.025**
***IRF7***					
rs34948036	11:612823	insT	22.22	40	40
*p*-value			Mild vs. moderate	Moderate vs. severe	Mild vs. severe
			**0.024**	1.000	**0.024**
rs1051390	11:613165	G>C	11.11	10.00	30.00
*p*-value			Mild vs. moderate	Moderate vs. severe	Mild vs. severe
			0.809	**0.001565**	**0.00322**
rs12422022	11:613192	A>G	11.11	10.00	30.00
*p*-value			Mild vs. moderate	Moderate vs. severe	Mild vs. severe
			0.809	**0.001565**	**0.00322**
rs1131665	11:613208	T>C	11.11	10.00	30.00
			Mild vs. moderate	Moderate vs. severe	Mild vs. severe
			0.809	**0.001565**	**0.00322**
***DDX58***					
rs10813831	9:32,526,146	G>A	11.11	10	25
*p*-value			Mild vs. moderate	Moderate vs. severe	Mild vs. severe
			0.809	**0.011**	**0.021**
rs1213032873	9:32,485,288	insA	0.00	0.00	10.00
*p*-value			Mild vs. moderate	Moderate vs. severe	Mild vs. severe
			N/A	**0.001565**	**0.001565**
***TMPRSS2***					
rs17854725	21:42845383	A>G	44.44	40.00	70.00
*p*-value			Mild vs. moderate	Moderate vs. severe	Mild vs. severe
			0.629	**0.004231**	**0.017**
rs73230068	21:42845167	G>C	0.00	10.00	15.00
*p*-value			Mild vs. moderate	Moderate vs. severe	Mild vs. severe
			**0.001565**	0.317	**0.000108**
***ACE2***					
rs2285666	X:15610348	C>T	15.00	25.00	5.00
*p*-value			Mild vs. moderate	Moderate vs. severe	Mild vs. severe
			0.114	**0.000261**	**0.025**

In order to adjust our analysis for age, sex, and comorbidities and taking into account the size of the dataset, we divided our participants into 1) groups of patients younger than 60 vs. 60 years old or older, 2) females vs. males, and 3) with vs. without comorbidities. This way, we could analyze if the frequency of appearance of genetic variants within each clinical symptom group will depend on any of these confounding factors. Our results (Supplementary Table S3) show that age, sex, and comorbidity differences do cause statistically significant differences in genetic variant frequencies within the study groups in many reported variants and genes of interest.

**FIGURE 2:**
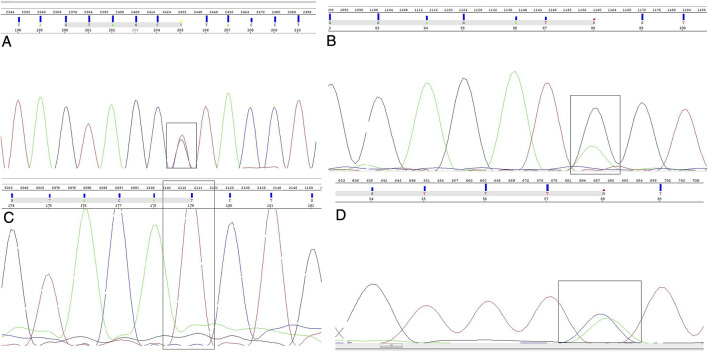
Sanger sequencing electropherograms. Nucleotide position of interest is shown in a black box. Nucleotides are stained as green—A, red—T, blue—C, and black—G. **(A)** Heterozygous genotype of *ACE2* rs2285666 polymorphism, **(B)** heterozygous genotype of *IFITM3* rs370862493 polymorphism, **(C)** homozygous mutant genotype of *TMPRSS2* rs12329760 polymorphism, and **(D)** heterozygous genotype of *IL4* rs2243290 polymorphism.

When it comes to confirmatory Sanger sequencing, done with the goal of validating the results of the custom-made NGS panel, we have re-sequenced five SNPs, namely rs2243290 C>A (*IL4* gene), rs370862493 G>A (*IFITM3* gene), rs2285666 C>T (*ACE2* gene), rs17854725 A>G, and rs12329760 C>T (both from *TMPRSS2* gene) (Figure 2). We obtained 100% agreement for the variants rs370862493, rs2285666, and rs12329760. As for 90% agreement between the methods for rs2243290 polymorphism on *IL4* gene, one sample from the severe symptom group was sequenced as homozygous variant using the Sanger method, while NGS reported it as a carrier of heterozygous genotype. When it comes to the *TMPRSS2* variant rs178854725, the designed primers for this variant could not be optimized for Sanger sequencing, since the bands on gel electrophoresis were acquired during PCR protocol optimization, but the sequencing results did not give clear, readable electropherograms.

## 4 Discussion

In the present study, we identified male sex, older age, and different classes of comorbidities as significant predictors of severe COVID-19. These findings were confirmed on extremely large cohorts, based on data from the US and Chinese Centers for Disease Control and Prevention (CDCs), whereby male sex and, especially, age of 50 years of life or older, were confirmed to be significantly associated with severe COVID-19, defined as the infection which requires hospitalization, ICU, or results in patient’s death. In addition, comorbidities were identified as an additional contributing factor to severe COVID-19, whereby 69.2% of all US patients had comorbidities compared to only 26.7% of Chinese patients, which can be explained by different definitions of comorbidities in these two countries (Zheng and Song, 2021).

We also detected 13 variants of interest dispersed among seven human genes, playing different roles in the immune system maintenance and viral binding and entry into the host cells, with functional significance in COVID-19 and, potentially, other viral infections. A summary of variant annotation for eQTL status and possible association with other conditions is given in Table 5.

**TABLE 5:** Variant annotation was performed in order to detect the type of polymorphism, the significance of association of the locus with the eQTL character using two resources, namely ELIXIR Estonia eQTL Catalogue Browser (Kerimov et al., 2021) and GTEx Portal (Carithers et al., 2015), as well as whether these variants are associated with any other conditions using ClinVar database within NCBI (Landrum et al., 2018). For the eQTL identification, we are giving only the annotated association with the lowest *p*-value and sample identity in which association were identified for both resources.

Gene	rs number	Type of polymorphism	Elixir Estonia eQTL	GTEx portal	Linked conditions
*CD55*	rs11120753	SNP	Naïve neutrophils, 1.03 × 10^−27^	Suprapubic skin, 3.6 × 10^−20^	Not reported
*IL1B*	rs1681980552	Deletion	No	No	Not reported
	rs1143634	SNP	No	Gastroesophageal junction, 0.0000041	Antisynthetase syndrome (association)
					Endometriosis (affects)
					Cholangiocarcinoma (other)
*IL4*	rs2243290	SNP	Naïve blood, 1.06 × 10^−7^	Tibial artery, 2.4 × 10^−25^	Not reported
*IRF7*	rs34948036	Insertion	CD8^+^ naïve T cells, 1.98 × 10^−9^	Thyroid, 6.0 × 10^−17^	Not reported
	rs1051390	SNP	Naïve DLPFC, 4.76 × 10^−17^	Tibial nerve, 6.6 × 10^−23^	Not reported
	rs12422022	SNP	4.76 × 10^−17^	Tibial nerve, 6.6 × 10^−23^	Not reported
	rs1131665	SNP	4.76 × 10^−17^	Tibial nerve, 6.6 × 10^−23^	Not reported
*DDX58*	rs10813831	SNP	No	Tibial nerve, 8.6 × 10^−14^	Not reported
	rs1213032873	Insertion	No	No	Not reported
*TMPRSS2*	rs17854725	SNP	No	Lung, 0.0000029	Not reported
	rs73230068	SNP	No	No	Not reported
*ACE2*	rs2285666	SNP	No	Lower leg skin, 2.8 × 10^−11^	Not reported

DLPFC, dorsolateral prefrontal cortex.

*TMPRSS2* and *ACE2* gene products are necessary for viral invasion of the host cells, which is why their variants are heavily researched and expected to be associated with COVID-19 severity, as well as susceptibility (Hou et al., 2020; Paniri et al., 2021). *TMPRSS2* is one of the main discoveries in understanding the mechanism of SARS-CoV-2 infection, as it codes for a cell-surface protein expressed by epithelial cells of different tissues, including the aerodigestive tract. SARS-CoV-2 entry into the host cells is dependent upon *TMPRSS2* since viral S glycoprotein is cleaved by TMPRSS2, which helps with viral activation (Hoffmann et al., 2020). *ACE2* is also crucial in SARS-CoV-2 infection, since the viral entry into the cell depends on ACE2 receptor, which can be found in respiratory tract, oral mucosa and heart cells (Aguiar et al., 2020). Kuba et al. (2005) found that expression of *ACE2* gene is downregulated in cells infected by SARS-CoV (Kuba et al., 2005). It was speculated that the genetic variants and loss-of-function mutations in *ACE2* might confer resistance to COVID-19, while hypomorphic variants of this gene could be protective against severe cases of COVID-19 disease (Casanova et al., 2020). There is evidence of sex-specific differences in the COVID-19 severity (Hou et al., 2020). For example, higher testosterone level increases the expression of *TMPRSS2*, which may cause higher susceptibility to COVID-19 in male patients (Bennani and Bennani-Baiti, 2020).

rs73230068 (G>C) is a single nucleotide change in the intronic region of *TMPRSS2*, which was present in five of our patients, whereby three of them belong to severe and two to moderate clinical symptoms group; all five individuals are heterozygous carriers. Our study, therefore, shows an increase in variant frequency in patients with more severe forms of COVID-19. While it was not a subject of previous research aiming to associate this variant with any diseases or clinical conditions, it has been a subject of a population study. Alternative allele frequency of 0.037 was recorded in 14,286 individuals of European ancestry (Phan et al., 2020), which is in good agreement with our allele frequency of 0.042 (*p* = 0.904) in a set of COVID-19 patients. rs17854725 A>G is a silent variant (c.879T>C, p.Ile293=) in the same gene, which is present in a significantly higher proportion of patients from the severe, when compared to mild and moderate symptom groups. The same variant is also present as a missense variant (c.879T>G, p.Ile293Met), which was not recorded in our study. This variant was previously investigated in terms of its association with COVID-19. Namely, rs17854725/rs75603675/rs12329760/rs4303795 polymorphisms have been associated with increased susceptibility to COVID-19 and more severe clinical symptoms (Rokni et al., 2022). In that study, mortality was more frequent in individuals who carried the rs17854725/AG genotype. They also showed that G allele of this SNP is related to increased susceptibility to COVID-19 infection. Combined haplotype rs17854725/AG, rs75603675/AC, rs12329760/TT, and rs4303795/AG was ruled as a risk factor for COVID-19 susceptibility, especially in the case of GATG and GCTG haplotypes. Most COVID-19 patients whose rs17854725 genotype was AG were affected by the severe form of the disease, while about 64% of the AA genotype carriers had mild clinical symptoms (Rokni et al., 2022). Additionally, in a bioinformatic prediction study (Paniri et al., 2021), rs75603675 was predicted to affect TMPRSS2 protein function according to PolyPen-2, but not according to SIFT (Paniri et al., 2021). Our study showed the presence of three polymorphisms together, that is, rs17854725/rs75603675/rs12329760 in two patients, whereby one patient belongs to mild and the other belongs to severe clinical symptoms group.

rs2285666 (C>T) intronic variant on X-chromosomal gene *ACE2* was detected in nine samples in our study, including one male from the severe clinical symptoms group, five patients from the moderate group (three heterozygous females and two males), and three patients from the mild group (two heterozygous females and one male). rs2285666 polymorphism is located at the beginning of the intron 3, and it could theoretically affect gene expression with alternative splicing mechanisms (Yang et al., 2015). Srivastava et al. (2020) made a correlation between lower SARS-CoV-2 infection rate and the minor allele (T) in Indian population, therefore establishing a possibility of this polymorphism being associated with a protective role against infection (Srivastava et al., 2020). Möhlendick et al. (2021) have found a two-fold increased risk of SARS-CoV-2 infection and a three-fold increased risk for COVID-19-related fatality or severe form of COVID-19 in CC genotype (or C allele) carriers in German population (Möhlendick et al., 2021). Similarly, a meta-analysis reported GG carriers as individuals at risk of developing severe COVID-19 (Saengsiwaritt et al., 2022). Nonetheless, it is important to note that different studies report conflicting results regarding this polymorphism. A more recent meta-analysis of 11 studies reports rs2285666 as associated with more severe COVID-19 (Aziz and Islam, 2022). Also, T allele was identified as a risk factor for severe or fatal COVID-19, especially in males, regardless of age, hypertension, T2DM, and obesity (Martinez-Gomez et al., 2022). Our study shows that there is an increased number of alternative allele carriers in mild and moderate groups, when compared to the group of patients with severe form of COVID-19, meaning that our results corroborate the hypothesis of this SNP being more prevalent in mild and moderate clinical groups. However, this is not necessarily the case when considering the sex of the study participants. Males, carrying one alternative allele and having one copy of the gene, are distributed across three groups. Females are all heterozygous, which is important considering the fact that *ACE2* escapes X chromosome inactivation (Gagliardi et al., 2020) and both copies of the gene remain transcriptionally active in all cells of female patients. This gives higher gene dosage to females, as well as evolutionary advantage in case of heterozygous carriers of harmful variants. This SNP, however, seems to be protective in females since it is found in moderate and mild groups only.

*CD55* variant rs11120753 (G>A) is reported in seven patients in our study, including one heterozygous carrier in severe symptom group, two homozygotes in moderate, and four patients from the mild symptom group, including three homozygous and one heterozygous carrier. According to the ALFA Project results (Phan et al., 2020), obtained from 17,796 individuals of European ancestry, alternate allele A is present with the frequency of 0.2697, which is higher when compared to our study population with allele frequency of 0.125, but the difference is not significant (*p* = 0.258). This is an intronic variant and there have been no previously published data on this variant regarding COVID-19 disease involvement. However, CD55 was found to be upregulated on the surface of monocytes in COVID-19 patients when compared to healthy controls (Lage et al., 2022), especially in lung tissue (Ge et al., 2023). *In silico* microarray data analysis from the ArrayExpress database revealed possible involvement of *CD55* differential expression in COVID-19 (Vastrad et al., 2020). Since CD55 is a cell-surface-bound glycoprotein acting as a complement inhibitor, its overexpression is suggested to play a role in self-protection due to complement overactivation in case of viral infection and further prevention of host cell damage.

Intronic variant rs1681980552 (delAAA) in *IL1B* gene was detected in nine patients. Five patients belong to the severe clinical group (two homozygotes and three heterozygotes), two to the moderate (one homozygote and one heterozygote), and two to the mild clinical group (both heterozygous carriers). There are no reports on this variant for any disease association and there are no population studies on its frequency, but we are reporting it as a promising target for predicting possible severe symptoms of COVID-19. Polymorphism rs1143634 (G>A) from the same gene is detected in nine patients, including six patients from the severe, one patient from the moderate, and two patients from the mild clinical group, whereby all variant alleles were detected in heterozygous genotypes. This is a synonymous variant (p.Phe105=) reported in ClinVar (Landrum et al., 2018) as associated with antisynthetase syndrome and endometriosis. Jafrin, Aziz and Islam (2021) performed a meta-analysis which revealed that the presence of this polymorphism increases the risk of cancer development, more precisely gastric and breast cancers and multiple myeloma, especially in Asian populations (Jafrin et al., 2021). Several studies connect rs1143634 with chronic periodontitis, including a meta-analysis by da Silva et al. (2017), in which it was significantly associated with chronic periodontitis disease in Caucasian, Asian and mixed populations (da Silva et al., 2017). There is no reported data on this variant regarding its association with SARS-CoV-2 infection or the clinical course of the disease, prior to our study in which this variant seems to be overrepresented in the severe symptom group when compared to the other two. *IL1B* is an inflammatory cytokine involved in initiating the immunological response against viral infection and is therefore highly relevant for viral infections. Previous studies reported that *IL1B* deregulation could be among the causes of cytokine storm and critical and/or severe COVID-19 symptoms (Chua et al., 2020; Lee et al., 2020; Feng et al., 2022), as well as its high plasma levels in patients with post-acute sequelae of COVID-19 (PASC) (Schultheiss et al., 2022).

rs2243290 (C>T) is an intronic variant of *IL4* gene and it has been detected in 15 patients in our study (six from severe, six from moderate and three form mild clinical group). Just like other variants on genes encoding for interleukins, this variant is enriched in the study groups with more pronounced COVID-19 symptoms and, therefore, might be associated with disease severity. This variant was not previously reported in relation to COVID-19; however, *IL4* has been studied regarding susceptibility to SARS-CoV infection, and it was found that its protein product downregulates cell surface expression of ACE2, therefore inhibiting SARS-CoV replication (de Lang et al., 2006). This gene and its protein product were reported in relation to COVID-19, as IL4 is generally activated interleukin in bodily immune response to SARS-CoV-2 infection (Hasanvand, 2022). Increased IL4 serum levels were associated with patients with previous infection without signs of long COVID-19 (Queiroz et al., 2022), but also with lung tissue samples from COVID-19 patients who did not survive the infection (Vaz de Paula et al., 2021). Since individuals with asthma and allergic diseases, which are not commonly encountered comorbidities in COVID-19, experience overactivation of type 2 immune response, including IL4, it is also proposed that the overexpression of this protein might play a protective role against COVID-19 infection (Liu et al., 2020; Gao et al., 2022).

rs34948036 (insT) is an intronic variant from *IRF7* gene detected in 16 patients, four of them belonging to mild, four to moderate, and eight to severe clinical symptom group. Its alternative allele (insT) frequency in European population is 0.259, based on the ALFA Project (Phan et al., 2020) on 24,292 individuals. Our results show, for the first time, that this single-nucleotide insertion could be clinically relevant and associated with severe clinical symptoms of COVID-19. rs1051390 (G>C), rs12422022 (A>G), and rs1131665 (T>C) variants, also on *IRF7* gene, were detected in nine patients with identical distribution, as a haplotype. Six of these patients belong to the severe clinical symptoms group, one belongs to moderate, and two belong to mild symptoms group, whereby all nine participants presented with heterozygous genotype. While rs1051390 and rs12422022 are intronic, rs1131665 is a missense variant (g.613208T>C, p.Gln412Arg). Despite none of these variants being previously reported for COVID-19 association, *IRF7* gene codes for protein necessary to produce IFN-I. Autosomal recessive *IRF7* deficiency was reported in three patients with COVID-19 pneumonia symptoms, whereby *IRF7*-deficient patients are generally more prone to viral infection of the respiratory tract (Campbell et al., 2022). Additionally, type I interferon immunity deregulation due to IRF7 deficiency was suggested as a possible molecular mechanism of severe and life-threatening COVID-19 (Zhang et al., 2020).

rs10813831 (G>A) is a missense variant (g.5177C>T, p.Arg7Cys) in *DDX58* gene, that was detected in eight patients in our study, including five from the severe clinical group (one heterozygous and four homozygous genotypes), one heterozygote from the moderate clinical group, and two heterozygotes from the mild clinical group. Our results point towards its involvement in progression of more severe COVID-19, especially in homozygous carriers of the variant, which was recently confirmed in an Iranian study of 182 patients with mild and 177 with severe COVID-19 were genotyped for this polymorphism, whereby AA genotype was significantly associated with severe COVID-19 when compared to GG, in a recessive model (Feizollahi et al., 2023). Previous research connected this variant with other conditions as well. For example, Wu et al. (2019) concluded that Chinese individuals carrying the rs10813831-G-allele-containing genotype were more liable to achieve spontaneous hepatitis C virus (HCV) clearance than the patients who were carriers of the alternate allele (Wu et al., 2019). Another interesting variant from *DDX58* gene is rs1213032873 (insA), which is detected in only two heterozygous patients in the severe symptom group. It is an intronic variant with no clinical significance described in ClinVar (Landrum et al., 2018), including no reports on the variant association with COVID-19 susceptibility and/or severity. This insertion is extremely rare, with alternative allele frequency of 0.0002 in 8,676 individuals of European ancestry, according to the latest release of the ALFA Project (Phan et al., 2020), as compared to our allele frequency of 0.021 in the COVID-19 patient population, which is significantly different (*p* = 5.31 × 10^−16^). Since we are reporting this variant for the first time, to the best of our knowledge, as the variant potentially associated with severe COVID-19 manifestation and increased susceptibility to COVID-19, it should be researched on a large patient population and compared to the general population frequencies of the insertion allele. *DDX58* gene, a carrier of these two variants, also known as RNA sensor RIG-I, is involved in viral double-stranded RNA recognition and antiviral immune response in host cells. It has been reported that *DDX58* gene expression under SARS-CoV-2 infection is upregulated (Fricke-Galindo and Falfan-Valencia, 2021).

Peripheral blood mononuclear cell (PBMC) immunophenotyping proved to be an additional highly informative tool of analysis when it comes to distinguishing between the severity of COVID-19 presentation, as well as viral persistence in infected individuals. A previous study performed PBMC immunophenotyping using single-cell RNA sequencing (scRNA-seq) technique on 11 healthy controls, five asymptomatic infected individuals and 33 symptomatic patients of different clinical presentations of the disease. They came up with 76 different immune cell subsets, some of which were found to be significantly more common in asymptomatic than symptomatic patients, while others were associated with more or less severe disease course, as well as capable of modulating the extent of viral presence in infected cells. For example, (TRAV1-2^+^CD8^+^) MAIT cells, (NCAM1^hi^CD160^+^) NK cells, (CD4^lo^CSF1R^−^CD33^−^CD14^+^) classical monocytes, and (CD33^−^ HLA-DMA^-^CD14^+^) classical monocytes were associated with asymptomatic infection. It was also shown that (CD68^−^CSF1R^−^IL1B^hi^CD14^+^) classical monocytes were positively associated with more severe COVID-19 presentation, but potentially also with the disease progression mechanism. Additionally, *IL1B* and *IFITM3* were found to be upregulated in these cells in patients with severe COVID-19, when compared to healthy or asymptomatic individuals or patients with mild disease (Wang et al., 2022). Another study assessed 40 healthy individuals and 97 COVID-19 patients with different disease severity presentation and generated a dataset of 1,400 plasma proteins and 2,600 single-cell immune features to study the most commonly deregulated pathways during the progression of SARS-CoV-2 infection in the human body. It was found that JAK-STAT, MAPK-mTOR and NF-κB signaling pathways are deregulated in COVID-19 and might be used as early-stage predictors of COVID-19 severity. In addition, this study identified association of CD4 and CD8 T cell emergence in case of progression towards more severe COVID-19, as well as multiple proteome-level changes, such as RAS, lung homeostasis and hemostasis pathways enrichment, and cytokine storm elements, such as increased plasma levels of IL1B, IL-33, IL-6, and IFNγ. As an element of RAS system, increased ACE2 plasma levels were positively correlated with more severe COVID-19, which points towards its possible shedding from the cell surface and subsequent loss-of-function, which corroborates with expected increase in rate of cardiovascular damage and multi-organ injuries commonly seen in patients with severe COVID-19. An increased percentage of granulocytes in PBMC samples of patients with severe COVID-19 was also observed (Feyaerts et al., 2022). Another study compared immunophenotypic profiles of PBMCs and bronchoalveolar lavage fluid mononuclear cells (BALF-MCs) in 18 patients with COVID-19-associated acute respiratory distress syndrome (CARDS). The results showed similar profiles for these two types of cells in survivors and deceased patients. It was, however, reported that the frequency of classical monocytes and naïve CD4^+^ T cells in peripheral blood was higher in survivors than in non-survivors. Several differences were also detected between PBMCs and BALF-MCs, pointing towards specificities of disease progression in analyzed patients (Santinelli et al., 2023).

Genome-wide association study (GWAS) efforts are also an important source of information for COVID-19, especially since those studies are usually multicenter and based on large patient cohorts with matched controls. COVID Human Genetic Effort group studied 659 patients of different ancestries via whole-exome sequencing, as well as 534 controls with asymptomatic or mild clinical presentation. They detected 113 variants at 12 loci in the severely affected group, including nine predicted loss-of-function variants and 109 missense or in-frame indels, without copy-number variations. Significantly higher frequency of damaging loss-of-function or highly hypomorphic variants was identified in the severe group, including 24 rare nonsynonymous variants from eight genes, namely *TLR3, IRF3, IRF7, IFNAR1, IFNAR2, TBK1, TICAM1 (TRIF),* and *UNC93B1* (Zhang et al., 2022). COVID-19 Host Genetics Initiative published their findings on 46 studies performed in 19 countries including a total of 49,562 COVID-19 cases and two million ancestry-matched controls, with the goal of identifying genomic loci associated with COVID-19 susceptibility and severity. Overall, 13 significant loci were detected, with four of these loci being associated with increased susceptibility, including *ABO* locus, 3p21.31 genomic region (rs2271616 from *SLC6A20* gene), and *PPP1R15A*. Nine positions are linked to increased likelihood of more severe symptoms and worse clinical outcome, including variants in genes *TYK2, DPP9,* and *CXCR6* (Niemi et al., 2021; COVID-19 Host Genetics Initiative, 2020). Genetics of Mortality in Critical Care (GenOMICC) conducted a study on 2,244 critically ill patients with COVID-19 infection hospitalized in 208 intensive care units throughout the United Kingdom, along with five ancestry-matched controls from the United Kingdom Biobank for each case. The strongest association was found on chromosome 3 (rs73064425, closest to *LZTFL1*), but several other loci were found significantly associated with critical illness in COVID-19, such as those in the proximity to genes *OAS1, OAS2, OAS3, TYK2, DPP9,* and *IFNAR2*. In addition, it was found that *IFNAR2* underexpression, as well as *TYK2* overexpression are associated with critical illness, just like the differential expression in lung tissue of *CCR2, CCR3*, *CXCR6,* and *MTA2B* in critical disease (Pairo-Castineira et al., 2021). The Severe COVID-19 GWAS group included 835 patients and 1,255 controls in Italy and 775 patients and 950 controls from Spain, to study the genetics of severe presentation of COVID-19. The strongest association with severe COVID-19 was observed for genomic locus 3p21.31 covering genes *SLC6A20, LZTFL1, CCR9, FYCO1, CXCR6* and *XCR1*. Another signal was detected from 9q34.2, that overlaps with the *ABO* blood group locus. Subsequent *HLA* locus analysis did not reveal any SNPs associated with either susceptibility to COVID-19 or with disease severity (Ellinghaus et al., 2020).

Considering all previously presented findings and literature sources, it is important to identify the limitations of the present study. Firstly, our limited sample size does not permit us to draw any final conclusions related to the functional significance of detected variants. We are reporting these variants with recommendations for their future testing and analysis in order to fully understand their role, not only in COVID-19, but in other viral infections as well, in terms of both susceptibility and disease progression. Additional testing methods, including transcriptomics and proteomics analyses, flow cytometry for immunophenotyping, as well as WGS and WES sequencing would provide more comprehensive results and enable us to get a full picture of the molecular effect of detected variants and the mechanism of their action.

COVID-19 is probably the best representation of how significant discoveries can be made in short time periods, assuming that research teams are given adequate support. By studying COVID-19 from different perspectives, we accumulated a significant wealth of knowledge in less than 4 years since the beginning of the pandemic. Research into the host genetics of SARS-CoV-2 infection is still active and ongoing, with large consortia being formed with the goal of completing whole-genome or whole-exome studies on large patient and control groups. Despite being limited by small sample size and smaller portions of the genome being analyzed, this study also contributes to the present state of knowledge related to COVID-19, as it provides the only and unique look into the Western Balkans populations in relation to this infection. Apart from studying the influence of host genetics on COVID-19 susceptibility and severity, international research groups aim towards better understanding of critical COVID-19 and long-COVID, as well as how these findings can be used in future for better understanding and management of other viral infections in humans. Overall, given the current importance of this topic and availability of technical support, COVID-19 research promises to be one of the most fruitful areas of research in foreseeable future.

## Data Availability

The datasets presented in this study can be found in online repositories. The names of the repository/repositories and accession number(s) can be found below: https://www.ncbi.nlm.nih.gov/bioproject/?term=912097.
