# Supplementary material for: Identification of human genetic variants modulating the course of COVID-19 infection with importance in other viral infections
Source: Front Genet. 2023 Aug 29;14:1240245. doi: 10.3389/fgene.2023.1240245 (PMC10545899; doi:10.3389/fgene.2023.1240245)
Supplement: Supplementary file 1 [file Table1.DOCX]

Supplementary Material

**Identification of Human Genetic Variants Modulating the Course of**

**COVID-19 Infection with Importance in Other Viral Infections**

Lana Salihefendić^1,2^, Ivana Čeko^1,2^, Larisa Bešić^2^, Naida Mulahuseinović^1^, Selma Durgut^1^, Dino Pećar^1^, Lejla Prnjavorac^3^, Enis Kandić^1^, Neven Meseldžić^4^, Tamer Bego^4^, Besim Prnjavorac^3^, Damir Marjanović^2,5^, Rijad Konjhodžić^1,2^, Adna Ašić^2,*^

^1^ALEA Genetic Center, Sarajevo 71000, Bosnia and Herzegovina

^2^Department of Genetics and Bioengineering, International Burch University, Ilidža 71210, Sarajevo, Bosnia and Herzegovina

^3^General Hospital Tešanj, Tešanj 74260, Bosnia and Herzegovina

^4^Department of Pharmaceutical Biochemistry and Laboratory Diagnostics, Faculty of Pharmacy, University of Sarajevo, Sarajevo 71000, Bosnia and Herzegovina

^5^Institute for Anthropological Research, University of Zagreb, Zagreb 10000, Croatia

***Correspondence:**Adna Ašić
adna.asic@ibu.edu.ba

# Supplementary Tables

**Supplementary Table 1.** The list of all detected genetic polymorphisms with p-values expressing the differences in frequency of appearance between the study groups. Frequencies are given as percentage of all tested patients per study group in which variant was detected, regardless of genotype. p-values denoting statistically significant differences between the study groups are bolded.

| **rs number** | **Position** | **Change** | **Mild symptom group (%)** | **Moderate symptom group (%)** | **Severe symptom group (%)** |
| --- | --- | --- | --- | --- | --- |
| ***CD55*** | | | | | |
| rs11120753 | 1:207527285 | G>A | 22.2 | 20 | 5 |
| p-value | | | Mild vs. moderate | Moderate vs. severe | Mild vs. severe |
|  |  |  | 0.735 | **0.0027** | **0.000974** |
| rs10746463 | 1:207510596 | G>A | 44.4 | 30 | 55 |
| p-value | | | Mild vs. moderate | Moderate vs. severe | Mild vs. severe |
|  |  |  | 0.095 | **0.006695** | 0.288 |
| rs1507757 | 1:207504748 | C>A | 38.9 | 30 | 45 |
| p-value | | | Mild vs. moderate | Moderate vs. severe | Mild vs. severe |
|  |  |  | 0.284 | 0.083 | 0.505 |
| rs2184476 | 1:207504669 | G>A | 38.9 | 20 | 45 |
| p-value | | | Mild vs. moderate | Moderate vs. severe | Mild vs. severe |
|  |  |  | **0.014** | **0.00193** | 0.505 |
| rs762199546 | 1:207513719 | delT | 0 | 10 | 5 |
| p-value | | | Mild vs. moderate | Moderate vs. severe | Mild vs. severe |
|  |  |  | **0.001565** | 0.197 | **0.025** |
| ***IL1B*** | | | | | |
| rs1143633 | 2:113590467 | C>T | 11.111111 | 50 | 30 |
| p-value | | | Mild vs. moderate | Moderate vs. severe | Mild vs. severe |
|  |  |  | **6.54 x 10^-7^** | **0.025** | **0.00322** |
| rs1143639 | 2:113588793 | C>T | 15 | 10 | 15 |
| p-value | | | Mild vs. moderate | Moderate vs. severe | Mild vs. severe |
|  |  |  | 0.317 | 0.317 | 1 |
| rs1681980552 | 2:113588756 | delAA | 10 | 10 | 25 |
| p-value | | | Mild vs. moderate | Moderate vs. severe | Mild vs. severe |
|  |  |  | 1 | **0.011** | **0.011** |
| rs1143634 | 2:113590390 | G>A | 11.11111 | 10 | 30 |
| p-value | | | Mild vs. moderate | Moderate vs. severe | Mild vs. severe |
|  |  |  | 0.809 | **0.001565** | **0.00322** |
| rs1681980552 | 2:113588756 | C>A | 5 | 5 | 0 |
| p-value | | | Mild vs. moderate | Moderate vs. severe | Mild vs. severe |
|  |  |  | 1 | **0,025** | **0.025** |
| rs16062 | 2:113591081 | G>A | 0 | **0** | **5** |
| p-value | | | Mild vs. moderate | Moderate vs. severe | Mild vs. severe |
|  |  |  | N/A | **0.025** | **0.025** |
| ***DDX58*** | | | | | |
| rs10813831 | 9:32526146 | G>A | 11.11 | 10 | 25 |
| p-value | | | Mild vs. moderate | Moderate vs. severe | Mild vs. severe |
|  |  |  | 0.809 | **0.011** | **0.021** |
| rs1213032873 | 9:32485288 | insA | 0.00 | 0.00 | 10.00 |
| p-value | | | Mild vs. moderate | Moderate vs. severe | Mild vs. severe |
|  |  |  | N/A | **0.001565** | **0.001565** |
| rs45589431 | 9:32466277 | C>T | 20 | 25 | 20 |
| p-value | | | Mild vs. moderate | Moderate vs. severe | Mild vs. severe |
|  |  |  | 0.456 | 0.456 | 1 |
| rs3205166 | 9:32459450 | T>G | 27.77777778 | 0 | 35 |
| p-value | | | Mild vs. moderate | Moderate vs. severe | Mild vs. severe |
|  |  |  | **1.36 x 10^-7^** | **3.30 x 10^-9^** | 0.362 |
| rs17289927 | 9:32492352 | T>C | 15 | 20 | 20 |
| p-value | | | Mild vs. moderate | Moderate vs. severe | Mild vs. severe |
|  |  |  | 0.398 | 1 | 0.398 |
| rs1337246090 | 9:32488237 | G>C | 33.33333 | 40 | 45 |
| p-value | | | Mild vs. moderate | Moderate vs. severe | Mild vs. severe |
|  |  |  | 0.436 | 0.588 | 0.187 |
| rs751946210 | 9:32466462 | G>T | 22.2222222 | 0 | 10 |
| p-value | | | Mild vs. moderate | Moderate vs. severe | Mild vs. severe |
|  |  |  | **2.43 x 10^-6^** | **0.001565** | **0.031** |
| rs13295938 | 9:32473088 | T>C | 5 | 0 | 0 |
| p-value | | | Mild vs. moderate | Moderate vs. severe | Mild vs. severe |
|  |  |  | **0.025** | N/A | **0.025** |
| rs376164723 | 9:32476960 | A>G | 5 | 0 | 0 |
| p-value | | | Mild vs. moderate | Moderate vs. severe | Mild vs. severe |
|  |  |  | **0.025** | N/A | **0.025** |
| rs17217280 | 9:32480251 | A>T | 22.2222222 | 0 | 15 |
| p-value | | | Mild vs. moderate | Moderate vs. severe | Mild vs. severe |
|  |  |  | **2.43 x 10^-6^** | **0.000108** | 0.237 |
| rs1337246090 | 9:32488237 | G>C | 33.3333333 | 40 | 40 |
| p-value | | | Mild vs. moderate | Moderate vs. severe | Mild vs. severe |
|  |  |  | 0.436 | 1 | 0.436 |
| rs10970987 | 9:32457189 | T>C | 15 | 5 | 10 |
| p-value | | | Mild vs. moderate | Moderate vs. severe | Mild vs. severe |
|  |  |  | **0.025** | 0.197 | 0.317 |
| rs148010596 | 9:32526172 | T>G | 0 | 10 | 0 |
| p-value | | | Mild vs. moderate | Moderate vs. severe | Mild vs. severe |
|  |  |  | **0.001565** | **0.001565** | N/A |
| rs61752945 | 9:32481339 | C>T | 0 | 5 | 0 |
| p-value | | | Mild vs. moderate | Moderate vs. severe | Mild vs. severe |
|  |  |  | **0.025** | **0.025** | N/A |
| rs72710678 | 9:32500832 | C>T | 0 | 5 | 0 |
| p-value | | | Mild vs. moderate | Moderate vs. severe | Mild vs. severe |
|  |  |  | **0.025** | **0.025** | N/A |
| rs3205166 | 9:32459450 | T>G | 27.77777778 | 0 | 30 |
| p-value | | | Mild vs. moderate | Moderate vs. severe | Mild vs. severe |
|  |  |  | **1.36 x 10^-7^** | **4.32 x 10^-8^** | 0.77 |
| rs752109554 | 9:32481500 | dupA | 0 | 0 | 5 |
| p-value | | | Mild vs. moderate | Moderate vs. severe | Mild vs. severe |
|  |  |  | N/A | **0.025** | **0.025** |
| ***TMPRSS2*** | | | | | |
| rs17854725 | 21:42845383 | A>G | 44.44 | 40.00 | 70.00 |
| p-value | | | Mild vs. moderate | Moderate vs. severe | Mild vs. severe |
|  |  |  | 0.629 | **0.004231** | **0.017** |
| rs73230068 | 21:42845167 | G>C | 0.00 | 10.00 | 15.00 |
| p-value | | | Mild vs. moderate | Moderate vs. severe | Mild vs. severe |
|  |  |  | **0.001565** | 0.317 | **0.000108** |
| rs55964536 | 21:42845638 | C>T | 55 | 60 | 45 |
| p-value | | | Mild vs. moderate | Moderate vs. severe | Mild vs. severe |
|  |  |  | 0.641 | 0.143 | 0.317 |
| rs75603675 | 21:42879909 | C>A | 20 | 30 | 25 |
| p-value | | | Mild vs. moderate | Moderate vs. severe | Mild vs. severe |
|  |  |  | 0.157 | 0.5 | 0.456 |
| rs764392003 | 21:42848561 | delaAG | 44.4444444 | 30 | 50 |
| p-value | | | Mild vs. moderate | Moderate vs. severe | Mild vs. severe |
|  |  |  | 0.094 | **0.025** | 0.568 |
| rs422471 | 21:42860307 | C>T | 33.33333333 | 40 | 35 |
| p-value | | | Mild vs. moderate | Moderate vs. severe | Mild vs. severe |
|  |  |  | 0.436 | 0.564 | 0.84 |
| rs73905370 | 21:42838138 | A>T | 30 | 10 | 15 |
| p-value | | | Mild vs. moderate | Moderate vs. severe | Mild vs. severe |
|  |  |  | **0.001565** | 0.317 | **0.025** |
| rs55896064 | 21:42838198 | G>A | 30 | 10 | 15 |
| p-value | | | Mild vs. moderate | Moderate vs. severe | Mild vs. severe |
|  |  |  | **0.001565** | 0.317 | **0.025** |
| rs2298660 | 21:42845633 | C>T | 30 | 10 | 40 |
| p-value | | | Mild vs. moderate | Moderate vs. severe | Mild vs. severe |
|  |  |  | **0.001565** | **0.000022** | 0.232 |
| rs12329760 | 21:42852497 | C>T | 60 | 25 | 50 |
| p-value | | | Mild vs. moderate | Moderate vs. severe | Mild vs. severe |
|  |  |  | **0.000147** | **0.003892** | 0.34 |
| rs743542 | 21:42840172 | G>A | 10 | 0 | 25 |
| p-value | | | Mild vs. moderate | Moderate vs. severe | Mild vs. severe |
|  |  |  | **0.001565** | **5.73 x 10-7** | **0.011** |
| rs2298659 | 21:42845374 | G>A | 50 | 0 | 30 |
| p-value | | | Mild vs. moderate | Moderate vs. severe | Mild vs. severe |
|  |  |  | **1.54 x 10^-12^** | **4.32 x 10^-8^** | **0.025** |
| rs140141551 | 21:42845220 | C>A | 5 | 0 | 0 |
| p-value | | | Mild vs. moderate | Moderate vs. severe | Mild vs. severe |
|  |  |  | **0.025** | N/A | **0.025** |
| rs429442 | 21:42861332 | C>T | 20 | 30 | 25 |
| p-value | | | Mild vs. moderate | Moderate vs. severe | Mild vs. severe |
|  |  |  | 0.157 | 0.5 | 0.456 |
| rs764392003 | 21:42848561 | G>A | 0 | 0 | 5 |
| p-value | | | Mild vs. moderate | Moderate vs. severe | Mild vs. severe |
|  |  |  | N/A | **0.025** | **0.025** |
| rs74423429 | 21:42848579 | G>A | 5.555555556 | 0 | 5 |
| p-value | | | Mild vs. moderate | Moderate vs. severe | Mild vs. severe |
|  |  |  | **0.018** | **0.025** | 0.864 |
| rs2298661 | 21:42845642 | C>A | 35 | 10 | 35 |
| p-value | | | Mild vs. moderate | Moderate vs. severe | Mild vs. severe |
|  |  |  | **0.000194** | **0.000194** | 1 |
| rs71951459 | 21:42842714 | insCTAAGGCCTCGGG | 5 | 0 | 0 |
| p-value | | | Mild vs. moderate | Moderate vs. severe | Mild vs. severe |
|  |  |  | **0.025** | N/A | **0.025** |
| rs144948620 | 21:42861545 | G>A | 5 | 0 | 5 |
| p-value | | | Mild vs. moderate | Moderate vs. severe | Mild vs. severe |
|  |  |  | **0.025** | **0.025** | 1 |
| rs200615061 | 21:42848494 | A>C | 0 | 10 | 5 |
| p-value | | | Mild vs. moderate | Moderate vs. severe | Mild vs. severe |
|  |  |  | **0.001565** | 0.197 | **0.025** |
| rs186734573 | 21:42852365 | C>T | 5 | 0 | 5 |
| p-value | | | Mild vs. moderate | Moderate vs. severe | Mild vs. severe |
|  |  |  | **0.025** | **0.025** | 1 |
| ***ACE2*** | | | | | |
| rs2285666 | X:15610348 | C>T | 15.00 | 25.00 | 5.00 |
| p-value | | | Mild vs. moderate | Moderate vs. severe | Mild vs. severe |
|  |  |  | 0.114 | **0.000261** | **0.025** |
| rs41297301 | X:15603508 | G>T | 5 | 10 | 5 |
| p-value | | | Mild vs. moderate | Moderate vs. severe | Mild vs. severe |
|  |  |  | 0.197 | 0.197 | 1 |
| rs35803318 | X:15582209 | C>T | 0 | 10 | 0 |
| p-value | | | Mild vs. moderate | Moderate vs. severe | Mild vs. severe |
|  |  |  | **0.001565** | **0.001565** | N/A |
| ***IFITM3*** | | | | | |
| rs34481144 | 11:320836 | C>T | 22.222222 | 10 | 20 |
| p-value | | | Mild vs. moderate | Moderate vs. severe | Mild vs. severe |
|  |  |  | **0.031** | 0.068 | 0.732 |
| rs201130624 | 11:320549 | C>G | 5 | 10 | 0 |
| p-value | | | Mild vs. moderate | Moderate vs. severe | Mild vs. severe |
|  |  |  | 0.197 | **0.001565** | **0.025** |
| rs12252 | 11:320772 | A>G | 16.66666667 | 0 | 5 |
| p-value | | | Mild vs. moderate | Moderate vs. severe | Mild vs. severe |
|  |  |  | **0.000045** | **0.025** | **0.012** |
| rs1136853 | 11:320805 | G>T | 16.66666667 | 0 | 5 |
| p-value | | | Mild vs. moderate | Moderate vs. severe | Mild vs. severe |
|  |  |  | **0.000045** | **0.025** | **0.012** |
| rs370862493 | 11:320007 | G>A | 0 | 0 | 5 |
| p-value | | | Mild vs. moderate | Moderate vs. severe | Mild vs. severe |
|  |  |  | N/A | **0.025** | **0.025** |
| ***IRF7*** | | | | |  |
| rs34948036 | 11:612823 | insT | 22.22 | 40 | 40 |
| p-value | | | Mild vs. moderate | Moderate vs. severe | Mild vs. severe |
|  |  |  | **0.024** | 1.000 | **0.024** |
| rs1051390 | 11:613165 | G>C | 11.11 | 10.00 | 30.00 |
| p-value | | | Mild vs. moderate | Moderate vs. severe | Mild vs. severe |
|  |  |  | 0.809 | **0.001565** | **0.00322** |
| rs12422022 | 11:613192 | A>G | 11.11 | 10.00 | 30.00 |
| p-value | | | Mild vs. moderate | Moderate vs. severe | Mild vs. severe |
|  |  |  | 0.809 | **0.001565** | **0.00322** |
| rs1131665 | 11:613208 | T>C | 11.11 | 10.00 | 30.00 |
| p-value | | | Mild vs. moderate | Moderate vs. severe | Mild vs. severe |
|  |  |  | 0.809 | **0.001565** | **0.00322** |
| rs534955005 | 11:613731 | C>T | 16.66666667 | 20 | 25 |
| p-value | | | Mild vs. moderate | Moderate vs. severe | Mild vs. severe |
|  |  |  | 0.582 | 0.456 | 0.197 |
| rs1061501 | 11:614864 | C>T | 27.777778 | 10 | 25 |
| p-value | | | Mild vs. moderate | Moderate vs. severe | Mild vs. severe |
|  |  |  | **0.002823** | **0.011** | 0.702 |
| rs1856512879 | 11:612843 | T>G | 11.1111111 | 20 | 25 |
| p-value | | | Mild vs. moderate | Moderate vs. severe | Mild vs. severe |
|  |  |  | 0.111 | 0.456 | **0.021** |
| rs11246213 | 11:612967 | A>G | 25 | 50 | 45 |
| p-value | | | Mild vs. moderate | Moderate vs. severe | Mild vs. severe |
|  |  |  | **0.003892** | 0.608 | **0.017** |
| rs1061505 | 11:613297 | T>G | 35 | 50 | 50 |
| p-value | | | Mild vs. moderate | Moderate vs. severe | Mild vs. severe |
|  |  |  | 0.104 | 1 | 0.104 |
| rs1061502 | 11:614318 | T>C | 35 | 55 | 45 |
| p-value | | | Mild vs. moderate | Moderate vs. severe | Mild vs. severe |
|  |  |  | **0.035** | 0.317 | 0.264 |
| rs12290989 | 11:615010 | G>T | 30 | 35 | 40 |
| p-value | | | Mild vs. moderate | Moderate vs. severe | Mild vs. severe |
|  |  |  | 0.535 | 0.564 | 0.232 |
| rs61877853 | 11:613697 | A>G | 5.5555556 | 0 | 10 |
| p-value | | | Mild vs. moderate | Moderate vs. severe | Mild vs. severe |
|  |  |  | **0.018** | **0.001565** | 0.26 |
| rs1856544445 | 11:613199 | C>T | 0 | 0 | 5 |
| p-value | | | Mild vs. moderate | Moderate vs. severe | Mild vs. severe |
|  |  |  | N/A | **0.025** | **0.025** |
| rs11246214 | 11:614367 | T>C | 0 | 30 | 10 |
| p-value | | | Mild vs. moderate | Moderate vs. severe | Mild vs. severe |
|  |  |  | **4.32 x 10^-8^** | **0.001565** | **0.001565** |
| rs113083699 | 11:615103 | G>A | 0 | 25 | 0 |
| p-value | | | Mild vs. moderate | Moderate vs. severe | Mild vs. severe |
|  |  |  | **5.73 x 10^-7^** | **5.73 x 10^-7^** | N/A |
| ***IL4*** | | | | | |
| rs2243290 | 5:132018169 | C>A | 15.00 | 30.00 | 30.00 |
| p-value | | | Mild vs. moderate | Moderate vs. severe | Mild vs. severe |
|  |  |  | **0.025** | 1.000 | **0.025** |
| rs2070874 | 5:132009710 | G>A | 25 | 25 | 40 |
| p-value | | | Mild vs. moderate | Moderate vs. severe | Mild vs. severe |
|  |  |  | 1 | 0.063 | 0.063 |
| rs35648164 | 5:132015555 | C>T | 0 | 5 | 0 |
| p-value | | | Mild vs. moderate | Moderate vs. severe | Mild vs. severe |
|  |  |  | **0.025** | **0.025** | N/A |
| rs2243251 | 5:132009787 | A>G | 0 | 0 | 5 |
| p-value | | | Mild vs. moderate | Moderate vs. severe | Mild vs. severe |
|  |  |  | N/A | **0.025** | **0.025** |
| ***TNF*α** | | | | | |
| rs3093664 | 6:31544642 | A>G | 5.555555556 | 0 | 5 |
| p-value | | | Mild vs. moderate | Moderate vs. severe | Mild vs. severe |
|  |  |  | **0.018** | **0.025** | 0.864 |
| ***IL1A*** | | | | | |
| rs17561 | 2:113537223 | C>A | 50 | 35 | 25 |
| p-value | | | Mild vs. moderate | Moderate vs. severe | Mild vs. severe |
|  |  |  | 0.104 | 0.197 | **0.003892** |
| rs3783550 | 2:113532885 | G>T | 38.88888889 | 10 | 40 |
| p-value | | | Mild vs. moderate | Moderate vs. severe | Mild vs. severe |
|  |  |  | **0.000036** | **0.000022** | 0.9 |
| rs1894399 | 2:113540177 | C>T | 22.22222222 | 10 | 20 |
| p-value | | | Mild vs. moderate | Moderate vs. severe | Mild vs. severe |
|  |  |  | **0.031** | 0.068 | 0.732 |
| ***IL6*** | | | | | |
| rs2069858 | 7:22767325 | G>A | 5.555555556 | 0 | 0 |
| p-value | | | Mild vs. moderate | Moderate vs. severe | Mild vs. severe |
|  |  |  | **0.018** | N/A | **0.018** |
| rs140764737 | 7:22767226 | G>A | 0 | 0 | 5 |
| p-value | | | Mild vs. moderate | Moderate vs. severe | Mild vs. severe |
|  |  |  | N/A | **0.025** | **0.025** |

**Supplementary Table 2.** Clinical exome sequencing results with all detected variants for three patients, including allele frequencies detected previously in European populations.

| **Sample 43** | | | | | | | | |
| --- | --- | --- | --- | --- | --- | --- | --- | --- |
| **Chr** | **Position** | | **Change** | **Genotype** | **Mutation type** | **Gene** | **Population frequency** | **Group** |
| chr7 | 22767226 | | G>A | Heterozygous | SNP | *IL6* | 0.002 | 1000 Genome European |
| chr7 | 22770846 | | delA | Heterozygous | DEL | *IL6* | 0.0194 | GnomAD Non-Finnish European |
| chr1 | 207492094 | | T>A | Heterozygous | SNP | *CD55* | - |  |
| chr1 | 207492413 | | G>A | Heterozygous | SNP | *CD55* | - |  |
| chr1 | 207511004 | | T>A | Heterozygous | SNP | *CD55* | - |  |
| chr1 | 207492170 | | delT | Heterozygous | DEL | *CD55* | 0.263 | GnomAD Non-Finnish European |
| chr1 | 207496129 | | C>T | Heterozygous | SNP | *CD55* | 0.2435 | 1000 Genome European |
| chr1 | 207500293 | | C>T | Heterozygous | SNP | *CD55* | 0.7445 | 1000 Genome European |
| chr1 | 207504669 | | G>A | Heterozygous | SNP | *CD55* | 0.7435 | 1000 Genome European |
| chr1 | 207504748 | | C>A | Heterozygous | SNP | *CD55* | 0.7435 | 1000 Genome European |
| chr1 | 207504820 | | delCA | Heterozygous | SNP | *CD55* | 0.7376 | 1000 Genome European |
| chr1 | 207509799 | | C>T | Heterozygous | SNP | *CD55* | 0.2545 | 1000 Genome European |
| chr1 | 207510289 | | T>C | Heterozygous | SNP | *CD55* | 1 |  |
| chr1 | 207510548 | | A>G | Heterozygous | SNP | *CD55* | 0.7445 | 1000 Genome European |
| chr1 | 207510596 | | G>A | Heterozygous | SNP | *CD55* | 0.7445 | 1000 Genome European |
| chr1 | 207532654 | | G>A | Homozygous | SNP | *CD55* | 1 |  |
| chr1 | 207532861 | | dupT | Heterozygous | DUP | *CD55* | 0.2479 | GnomAD Non-Finnish European |
| chr2 | 113586521 | | T>A | Heterozygous | SNP | *IL1B* | - |  |
| chr2 | 113589942 | | T>A | Heterozygous | SNP | *IL1B* | - |  |
| chr2 | 113592860 | | G>A | Heterozygous | SNP | *IL1B* | - |  |
| chr2 | 113592867 | | T>C | Heterozygous | SNP | *IL1B* | - |  |
| chr2 | 113594502 | | A>G | Heterozygous | SNP | *IL1B* | - |  |
| chr2 | 113588302 | | C>T | Heterozygous | SNP | *IL1B* | 0.338 | 1000 Genome European |
| chr2 | 113588553 | | A>G | Heterozygous | SNP | *IL1B* | 1 |  |
| chr2 | 113588756 | | delTT | Heterozygous | DEL | *IL1B* | 0.2445 | 1000 Genome European |
| chr2 | 113588793 | | C>T | Heterozygous | SNP | *IL1B* | 0.2445 | 1000 Genome European |
| chr2 | 113590390 | | G>A | Heterozygous | SNP | *IL1B* | 0.2475 | 1000 Genome European |
| chr2 | 113590467 | | C>T | Heterozygous | SNP | *IL1B* | 0.334 | 1000 Genome European |
| chr2 | 113591275 | | A>G | Heterozygous | SNP | *IL1B* | 0.2485 | 1000 Genome European |
| chr2 | 113591342 | | G>A | Heterozygous | SNP | *IL1B* | 0.1769 | 1000 Genome European |
| chr2 | 113592893 | | delT | Homozygous | DEL | *IL1B* | 0.2038 | 1000 Genome European |
| chr2 | 113593518 | | G>A | Homozygous | SNP | *IL1B* | 0.6471 | 1000 Genome European |
| chr21 | 42845994 | | G>A | Heterozygous | SNP | *TMPRSS2* | - |  |
| chr21 | 42846217 | | C>G | Heterozygous | SNP | *TMPRSS2* | - |  |
| chr21 | 42846218 | | A>G | Heterozygous | SNP | *TMPRSS2* | - |  |
| chr21 | 42846309 | | A>G | Heterozygous | SNP | *TMPRSS2* | - |  |
| chr21 | 42846321 | | G>A | Heterozygous | SNP | *TMPRSS2* | - |  |
| chr21 | 42846389 | | A>G | Heterozygous | SNP | *TMPRSS2* | - |  |
| chr21 | 42846401 | | G>A | Heterozygous | SNP | *TMPRSS2* | - |  |
| chr21 | 42847173 | | A>G | Heterozygous | SNP | *TMPRSS2* | - |  |
| chr21 | 42847182 | | A>G | Heterozygous | SNP | *TMPRSS2* | - |  |
| chr21 | 42847187 | | G>T | Heterozygous | SNP | *TMPRSS2* | - |  |
| chr21 | 42847192 | | A>G | Heterozygous | SNP | *TMPRSS2* | - |  |
| chr21 | 42847305 | | T>G | Heterozygous | SNP | *TMPRSS2* | 0 | GnomAD Non-Finnish European |
| chr21 | 42861902 | | T>C | Heterozygous | SNP | *TMPRSS2* | 0 | GnomAD Non-Finnish European |
| chr21 | 42861919 | | A>G | Homozygous | SNP | *TMPRSS2* | 0.0002 | GnomAD Non-Finnish European |
| chr21 | 42866825 | | G>T | Heterozygous | SNP | *TMPRSS2* | - |  |
| chr21 | 42832541 | | C>T | Heterozygous | SNP | *TMPRSS2* | 0.3728 | 1000 Genome European |
| chr21 | 42839038 | | G>A | Heterozygous | SNP | *TMPRSS2* | 0.1362 | 1000 Genome European |
| chr21 | 42839072 | | G>T | Homozygous | SNP | *TMPRSS2* | 0.9672 | 1000 Genome European |
| chr21 | 42839196 | | G>A | Homozygous | SNP | *TMPRSS2* | 1 | 1000 Genome European |
| chr21 | 42845374 | | G>A | Homozygous | SNP | *TMPRSS2* | **0.2296** | 1000 Genome European |
| chr21 | 42847036 | | A>G | Homozygous | SNP | *TMPRSS2* | 0.9801 | 1000 Genome European |
| chr21 | 42847185 | | G>C | Heterozygous | SNP | *TMPRSS2* | 0.3943 | GnomAD Non-Finnish European |
| chr21 | 42847212 | | G>A | Heterozygous | SNP | *TMPRSS2* | 0.413 | GnomAD Non-Finnish European |
| chr21 | 42847494 | | A>G | Heterozygous | SNP | *TMPRSS2* | 0.1235 | GnomAD Non-Finnish European |
| chr21 | 42847735 | | G>C | Heterozygous | SNP | *TMPRSS2* | 0.2306 | 1000 Genome European |
| chr21 | 42850075 | | T>C | Homozygous | SNP | *TMPRSS2* | 0.502 | 1000 Genome European |
| chr21 | 42852497 | | C>T | Homozygous | SNP | *TMPRSS2* | 0.2356 | 1000 Genome European |
| chr21 | 42854638 | | A>T | Homozygous | SNP | *TMPRSS2* | 0.4881 | 1000 Genome European |
| chr21 | 42859670 | | T>C | Heterozygous | SNP | *TMPRSS2* | 0.9781 | 1000 Genome European |
| chr21 | 42859876 | | C>T | Heterozygous | SNP | *TMPRSS2* | 0.7127 | 1000 Genome European |
| chr21 | 42865047 | | C>T | Heterozygous | SNP | *TMPRSS2* | 0.9761 | 1000 Genome European |
| chr21 | 42865324 | | G>A | Heterozygous | SNP | *TMPRSS2* | 0.7495 | 1000 Genome European |
| chr21 | 42866995 | | delT | Homozygous | SNP | *TMPRSS2* | 0.7651 | GnomAD Non Finnish European |
| chr21 | 42868997 | | A>C | Heterozygous | SNP | *TMPRSS2* | 0.1173 | 1000 Genome European |
| chr21 | 42869063 | | A>G | Heterozygous | SNP | *TMPRSS2* | 0.6511 | 1000 Genome European |
| chr21 | 42881166 | | G>A | Heterozygous | SNP | *TMPRSS2* | 0.3837 | 1000 Genome European |
| chr21 | 42881492 | | T>C | Homozygous | SNP | *TMPRSS2* | 0.8857 | 1000 Genome European |
| chr11 | 319211 | | C>T | Homozygous | SNP | *IFITM3* | - |  |
| chr11 | 321544 | | T>G | Heterozygous | SNP | *IFITM3* | - |  |
| chr11 | 322648 | | G>A | Heterozygous | SNP | *IFITM3* | - |  |
| chr11 | 322711 | | A>T | Heterozygous | SNP | *IFITM3* | - |  |
| chr11 | 320115 | | G>A | Heterozygous | SNP | *IFITM3* | 0.8519 | 1000 Genome European |
| chr11 | 320166 | | T>C | Heterozygous | SNP | *IFITM3* | 0.8519 | 1000 Genome European |
| chr11 | 320237 | | C>G | Heterozygous | SNP | *IFITM3* | 0.8874 | GnomAD Non-Finnish European |
| chr11 | 320394 | | C>T | Heterozygous | SNP | *IFITM3* | 0.4513 | 1000 Genome European |
| chr11 | 320836 | | C>T | Heterozygous | SNP | *IFITM3* | 0.4622 | 1000 Genome European |
| chr11 | 321044 | | G>A | Heterozygous | SNP | *IFITM3* | 0.3939 | 1000 Genome European |
| chr11 | 321055 | | G>T | Heterozygous | SNP | *IFITM3* | 0.3172 | 1000 Genome European |
| chr11 | 321104 | | G>A | Heterozygous | SNP | *IFITM3* | 0.2169 | GnomAD Non-Finnish European |
| chr11 | 321938 | | G>c | Heterozygous | SNP | *IFITM3* | 0.0089 | 1000 Genome European |
| chr11 | 325249 | | A>G | Heterozygous | SNP | *IFITM3* | 0.6759 | 1000 Genome European |
| chr11 | 613643 | | delTGTGAGTGACGGGGGTGGGCGGGG | Heterozygous | DEL | *IRF7* | 0.0175 | GnomAD Non-Finnish European |
| chr11 | 615647 | | C>A | Heterozygous | SNP | *IRF7* | - |  |
| chr11 | 612355 | | T>C | Heterozygous | SNP | *IRF7* | 0.2674 | 1000 Genome European |
| chr11 | 612843 | | T>G | Heterozygous | SNP | *IRF7* | 0.2674 | 1000 Genome European |
| chr11 | 612967 | | A>G | Heterozygous | SNP | *IRF7* | 0.2674 | 1000 Genome European |
| chr11 | 613165 | | G>C | Heterozygous | SNP | *IRF7* | 0.2674 | 1000 Genome European |
| chr11 | 613192 | | A>G | Heterozygous | SNP | *IRF7* | 0.2674 | 1000 Genome European |
| chr11 | 613208 | | T>C | Heterozygous | SNP | *IRF7* | 0.2674 | 1000 Genome European |
| chr11 | 613297 | | T>G | Heterozygous | SNP | *IRF7* | 0.2793 | 1000 Genome European |
| chr11 | 613697 | | A>G | Heterozygous | SNP | *IRF7* | 0.2058 | 1000 Genome European |
| chr11 | 613742 | | G>A | Heterozygous | SNP | *IRF7* | 0.2684 | 1000 Genome European |
| chr11 | 613757 | | A>G | Heterozygous | SNP | *IRF7* | 0.2694 | 1000 Genome European |
| chr11 | 614864 | | C>T | Homozygous | SNP | *IRF7* | 0.8748 | 1000 Genome European |
| chr11 | 615010 | | G>T | Heterozygous | SNP | *IRF7* | 0.2674 | 1000 Genome European |
| chr11 | 615011 | | A>T | Heterozygous | SNP | *IRF7* | 0.2674 | 1000 Genome European |
| chr5 | 132012399 | | A>C | Heterozygous | SNP | *IL4* | - |  |
| chr5 | 132009710 | | C>T | Heterozygous | SNP | *IL4* | 0.168 | 1000 Genome European |
| chr5 | 132011571 | | A>G | Heterozygous | SNP | *IL4* | 0.172 | 1000 Genome European |
| chr5 | 132011573 | | A>G | Heterozygous | SNP | *IL4* | 0.169 | 1000 Genome European |
| chr5 | 132013299 | | C>G | Heterozygous | SNP | *IL4* | 0.8976 | 1000 Genome European |
| chr5 | 132018132 | | A>G | Heterozygous | SNP | *IL4* | 0.168 | 1000 Genome European |
| chr5 | 132018169 | | C>A | Heterozygous | SNP | *IL4* | 0.17 | 1000 Genome European |
| chr2 | 113527079 | | T>C | Homozygous | SNP | *IL1A* | - |  |
| chr2 | 113530894 | | T>A | Heterozygous | SNP | *IL1A* | - |  |
| chr2 | 113535329 | | C>T | Heterozygous | SNP | *IL1A* | - |  |
| chr2 | 113535917 | | T>C | Heterozygous | SNP | *IL1A* | - |  |
| chr2 | 113538394 | | C>T | Heterozygous | SNP | *IL1A* | - |  |
| chr2 | 113532885 | | G>T | Homozygous | SNP | *IL1A* | 0.6779 | 1000 Genome European |
| chr2 | 113534830 | | G>C | Heterozygous | SNP | *IL1A* | 0.6779 | 1000 Genome European |
| chr2 | 113535438 | | C>T | Heterozygous | SNP | *IL1A* | 0.2873 | 1000 Genome European |
| chr2 | 113535771 | | delAC | Heterozygous | DEL | *IL1A* | 0.4264 | 1000 Genome European |
| chr2 | 113537223 | | C>A | Heterozygous | SNP | *IL1A* | 0.2873 | 1000 Genome European |
| chr2 | 113537339 | | A>G | Heterozygous | SNP | *IL1A* | 0.2873 | 1000 Genome European |
| chr2 | 113537579 | | T>C | Heterozygous | SNP | *IL1A* | 0.6789 | 1000 Genome European |
| chr2 | 113538490 | | A>G | Homozygous | SNP | *IL1A* | 0.6779 | 1000 Genome European |
| chr2 | 113540084 | | G>A | Heterozygous | SNP | *IL1A* | 0.6789 | 1000 Genome European |
| chr2 | 113540177 | | C>T | Heterozygous | SNP | *IL1A* | 0.2873 | 1000 Genome European |
| chr14 | 24633326 | | T>G | Heterozygous | SNP | *IRF9* | - |  |
| chr14 | 24633824 | | C>G | Heterozygous | SNP | *IRF9* | - |  |
| chr14 | 24636570 | | A>T | Heterozygous | SNP | *IRF9* | - |  |
| chr14 | 24637191 | | T>C | Heterozygous | SNP | *IRF9* | - |  |
| chr14 | 24637297 | | G>A | Heterozygous | SNP | *IRF9* | - |  |
| chr14 | 24638221 | | C>G | Heterozygous | SNP | *IRF9* | - |  |
| **Sample 49** | | | | | | | | |
| **Chr** | **Position** | **Change** | | **Genotype** | **Mutation type** | **Gene** | **Population Freq** | **Group** |
| chr1 | 207494701 | C>G | | Heterozygous | SNP | *CD55* | - |  |
| chr1 | 207498367 | T>A | | Heterozygous | SNP | *CD55* | - |  |
| chr1 | 207510466 | C>T | | Heterozygous | SNP | *CD55* | - |  |
| chr1 | 207532861 | insTT | | Heterozygous | INS | *CD55* | 0.2479 | GnomAD Non-Fin European |
| chr1 | 207495427 | G>T | | Homozygous | SNP | *CD55* | - |  |
| chr1 | 207498644 | C>T | | Homozygous | SNP | *CD55* | - |  |
| chr1 | 207500293 | C>T | | Homozygous | SNP | *CD55* | 0.7445 | 1000 Genomes European |
| chr1 | 207504669 | G>A | | Homozygous | SNP | *CD55* | 0.7435 | 1000 Genomes European |
| chr1 | 207504748 | C>A | | Homozygous | SNP | *CD55* | 0.7435 | 1000 Genomes European |
| chr1 | 207504820 | delAC | | Homozygous | DEL | *CD55* | 0.7376 | 1000 Genomes European |
| chr1 | 207510289 | T>C | | Homozygous | SNP | *CD55* | 1 | 1000 Genomes European |
| chr1 | 207510548 | A>G | | Homozygous | SNP | *CD55* | 0.7445 | 1000 Genomes European |
| chr1 | 207510596 | G>A | | Homozygous | SNP | *CD55* | 0.7445 | 1000 Genomes European |
| chr1 | 207532861 | insTTT | | Heterozygous | INS | *CD55* | 0.2479 | GnomAD Non-Fin European |
| chr1 | 207532862 | delTT | | Heterozygous | DEL | *CD55* | 0.0146 | GnomAD Non-Fin European |
| chr1 | 207532862 | del T | | Heterozygous | DEL | *CD55* | 0.0774 | GnomAD Non-Fin European |
| chr1 | 207535631 | T>C | | Homozygous | SNP | *CD55* | - |  |
| chr1 | 207538082 | T>C | | Homozygous | SNP | *CD55* | - |  |
| chr1 | 207539244 | G>A | | Homozygous | SNP | *CD55* | 1 | 1000 Genomes European |
| chr2 | 113589071 | A>G | | Heterozygous | SNP | *IL1B* | - |  |
| chr2 | 113590601 | T>A | | Heterozygous | SNP | *IL1B* | - |  |
| chr2 | 113591384 | A>T | | Heterozygous | SNP | *IL1B* | - |  |
| chr2 | 113585050 | C>T | | Heterozygous | SNP | *IL1B* | - |  |
| chr2 | 113585147 | T>C | | Heterozygous | SNP | *IL1B* | - |  |
| chr2 | 113588302 | C>T | | Heterozygous | SNP | *IL1B* | 0.338 | 1000 Genomes European |
| chr2 | 113590467 | C>T | | Heterozygous | SNP | *IL1B* | 0.334 | 1000 Genomes European |
| chr2 | 113593518 | G>A | | Heterozygous | SNP | *IL1B* | 0.6471 | 1000 Genomes European |
| chr2 | 113594867 | A>G | | Homozygous | SNP | *IL1B* | - |  |
| chr9 | 32500880 | G>A | | Heterozygous | SNP | *DDX58* | - |  |
| chr9 | 32459627 | C>A | | Heterozygous | SNP | *DDX58* | - |  |
| chr9 | 32464564 | T>C | | Homozygous | SNP | *DDX58* | - |  |
| chr9 | 32466056 | C>T | | Heterozygous | SNP | *DDX58* | - |  |
| chr9 | 32466061 | C>A | | Heterozygous | SNP | *DDX58* | - |  |
| chr9 | 32467518 | T>C | | Heterozygous | SNP | *DDX58* | - |  |
| chr9 | 32468202 | A>G | | Heterozygous | SNP | *DDX58* | - |  |
| chr9 | 32477922 | C>T | | Heterozygous | SNP | *DDX58* | - |  |
| chr9 | 32485289 | delA | | Heterozygous | DEL | *DDX58* | - |  |
| chr9 | 32492665 | A>G | | Heterozygous | SNP | *DDX58* | - |  |
| chr9 | 32506083 | G>T | | Heterozygous | SNP | *DDX58* | - |  |
| chr9 | 32506409 | C>A | | Heterozygous | SNP | *DDX58* | - |  |
| chr9 | 32512200 | T>C | | Heterozygous | SNP | *DDX58* | - |  |
| chr9 | 32457482 | delA | | Heterozygous | DEL | *DDX58* | - |  |
| chr9 | 32475904 | C>A | | Heterozygous | SNP | *DDX58* | - |  |
| chr9 | 32453278 | T>C | | Heterozygous | SNP | *DDX58* | - |  |
| chr9 | 32457558 | A>G | | Heterozygous | SNP | *DDX58* | - |  |
| chr9 | 32458940 | C>T | | Heterozygous | SNP | *DDX58* | - |  |
| chr9 | 32458964 | C>T | | Heterozygous | SNP | *DDX58* | - |  |
| chr9 | 32459450 | T>G | | Homozygous | SNP | *DDX58* | - |  |
| chr9 | 32462814 | G>T | | Heterozygous | SNP | *DDX58* | - |  |
| chr9 | 32463087 | C>T | | Heterozygous | SNP | *DDX58* | - |  |
| chr9 | 32466190 | T>C | | Homozygous | SNP | *DDX58* | - |  |
| chr9 | 32466277 | C>T | | Homozygous | SNP | *DDX58* | - |  |
| chr9 | 32466462 | G>T | | Homozygous | SNP | *DDX58* | - |  |
| chr9 | 32473088 | T>C | | Homozygous | SNP | *DDX58* | - |  |
| chr9 | 32480251 | A>T | | Homozygous | SNP | *DDX58* | - |  |
| chr9 | 32480592 | T>C | | Heterozygous | SNP | *DDX58* | - |  |
| chr9 | 32481500 | dupA | | Heterozygous | DUP | *DDX58* | - |  |
| chr9 | 32481601 | C>T | | Homozygous | SNP | *DDX58* | - |  |
| chr9 | 32484826 | A>G | | Homozygous | SNP | *DDX58* | 0.9284 | 1000 Genomes European |
| chr9 | 32484961 | G>T | | Homozygous | SNP | *DDX58* | - |  |
| chr9 | 32488557 | A>C | | Homozygous | SNP | *DDX58* | - |  |
| chr9 | 32491240 | C>T | | Homozygous | SNP | *DDX58* | 0.5577 | 1000 Genomes European |
| chr9 | 32492352 | T>C | | Homozygous | SNP | *DDX58* | - |  |
| chr9 | 32493620 | dupA | | Heterozygous | DUP | *DDX58* | - |  |
| chr9 | 32512287 | T>C | | Homozygous | SNP | *DDX58* | - |  |
| chr9 | 32525794 | insAG | | Homozygous | INS | *DDX58* | 0.6252 | 1000 Genomes European |
| chr9 | 32525969 | C>A | | Homozygous | SNP | *DDX58* | 0.6243 | 1000 Genomes European |
| chr9 | 32526233 | G>C | | Homozygous | SNP | *DDX58* | 0.6262 | 1000 Genomes European |
| chr21 | 42846241 | A>G | | Heterozygous | SNP | *TMPRSS2* | - |  |
| chr21 | 42846592 | C>T | | Heterozygous | SNP | *TMPRSS2* | - |  |
| chr21 | 42846629 | A>G | | Heterozygous | SNP | *TMPRSS2* | - |  |
| chr21 | 42847573 | A>G | | Heterozygous | SNP | *TMPRSS2* | 0 | GnomAD Non-Fin European |
| chr21 | 42850261 | C>T | | Heterozygous | SNP | *TMPRSS2* | - |  |
| chr21 | 42850285 | G>A | | Heterozygous | SNP | *TMPRSS2* | - |  |
| chr21 | 42864218 | G>T | | Heterozygous | SNP | *TMPRSS2* | - |  |
| chr21 | 42865830 | A>G | | Heterozygous | SNP | *TMPRSS2* | - |  |
| chr21 | 42860655 | C>T | | Homozygous | SNP | *TMPRSS2* | - |  |
| chr21 | 42839196 | G>A | | Homozygous | SNP | *TMPRSS2* | 1 | 1000 Genomes European |
| chr21 | 42839299 | C>G | | Homozygous | SNP | *TMPRSS2* | - |  |
| chr21 | 42841804 | C>T | | Heterozygous | SNP | *TMPRSS2* | - |  |
| chr21 | 42845383 | A>G | | Heterozygous | SNP | *TMPRSS2* | - |  |
| chr21 | 42845633 | C>T | | Heterozygous | SNP | *TMPRSS2* | - |  |
| chr21 | 42845638 | C>T | | Heterozygous | SNP | *TMPRSS2* | - |  |
| chr21 | 42845642 | C>A | | Heterozygous | SNP | *TMPRSS2* | - |  |
| chr21 | 42846184 | A>G | | Heterozygous | SNP | *TMPRSS2* |  |  |
| chr21 | 42846216 | G>A | | Heterozygous | SNP | *TMPRSS2* |  |  |
| chr21 | 42847036 | A>G | | Homozygous | SNP | *TMPRSS2* | 0.9801 | 1000 Genomes European |
| chr21 | 42847494 | A>G | | Heterozygous | SNP | *TMPRSS2* | 0.1235 | GnomAD Non-Fin European |
| chr21 | 42847735 | G>C | | Homozygous | SNP | *TMPRSS2* | 0.2306 | 1000 Genomes European |
| chr21 | 42855798 | T>C | | Heterozygous | SNP | *TMPRSS2* | - |  |
| chr21 | 42856048 | A>C | | Homozygous | SNP | *TMPRSS2* | - |  |
| chr21 | 42856116 | A>G | | Heterozygous | SNP | *TMPRSS2* | - |  |
| chr21 | 42859604 | G>A | | Homozygous | SNP | *TMPRSS2* | - |  |
| chrX | 15580809 | G>A | | Heterozygous | SNP | *ACE2* | - |  |
| chrX | 15595295 | A>G | | Homozygous | SNP | *ACE2* | - |  |
| chrX | 15582966 | G>A | | Heterozygous | SNP | *ACE2* | - |  |
| chrX | 15615453 | A>G | | Homozygous | SNP | *ACE2* | - |  |
| chr11 | 320289 | G>A | | Heterozygous | SNP | *IFITM3* | - |  |
| chr11 | 320953 | G>A | | Heterozygous | SNP | *IFITM3* | - |  |
| chr11 | 320115 | G>A | | Homozygous | SNP | *IFITM3* | 0.8519 | 1000 Genomes European |
| chr11 | 320166 | T>C | | Homozygous | SNP | *IFITM3* | 0.8519 | 1000 Genomes European |
| chr11 | 320237 | C>G | | Homozygous | SNP | *IFITM3* | 0.8874 | GnomAD Non-Fin European |
| chr11 | 320394 | C>T | | Homozygous | SNP | *IFITM3* | 0.4513 | 1000 Genomes European |
| chr11 | 320836 | C>T | | Heterozygous | SNP | *IFITM3* | 0.4622 | 1000 Genomes European |
| chr11 | 325249 | A>G | | Homozygous | SNP | *IFITM3* | 0.6759 | 1000 Genomes European |
| chr11 | 612355 | T>C | | Heterozygous | SNP | *IRF7* | 0.2674 | 1000 Genomes European |
| chr11 | 612822 | dupT | | Heterozygous | DUP | *IRF7* | - |  |
| chr11 | 612843 | T>G | | Heterozygous | SNP | *IRF7* | 0.2674 | 1000 Genomes European |
| chr11 | 612967 | A>G | | Heterozygous | SNP | *IRF7* | 0.2674 | 1000 Genomes European |
| chr11 | 613165 | G | | Heterozygous | SNP | *IRF7* | 0.2674 | 1000 Genomes European |
| chr11 | 613192 | A>G | | Heterozygous | SNP | *IRF7* | 0.2674 | 1000 Genomes European |
| chr11 | 613208 | T>C | | Heterozygous | SNP | *IRF7* | 0.2674 | 1000 Genomes European |
| chr11 | 613297 | T>G | | Heterozygous | SNP | *IRF7* | 0.2793 | 1000 Genomes European |
| chr11 | 613742 | G>A | | Heterozygous | SNP | *IRF7* | 0.2684 | 1000 Genomes European |
| chr11 | 613757 | A>G | | Heterozygous | SNP | *IRF7* | 0.2694 | 1000 Genomes European |
| chr11 | 614318 | T>C | | Heterozygous | SNP | *IRF7* | 0.2674 | 1000 Genomes European |
| chr11 | 614863 | C>T | | Homozygous | SNP | *IRF7* | - |  |
| chr11 | 615010 | G>T | | Heterozygous | SNP | *IRF7* | 0.2674 | 1000 Genomes European |
| chr11 | 615011 | A>T | | Heterozygous | SNP | *IRF7* | 0.2674 | 1000 Genomes European |
| chr5 | 132005774 | C>T | | Heterozygous | SNP | *IL4* | - |  |
| chr5 | 132005825 | G>A | | Heterozygous | SNP | *IL4* | - |  |
| chr5 | 132009482 | A>C | | Heterozygous | SNP | *IL4* | - |  |
| chr5 | 132011952 | A>G | | Heterozygous | SNP | *IL4* | - |  |
| chr5 | 132017999 | C>A | | Heterozygous | SNP | *IL4* | - |  |
| chr5 | 132006844 | G>C | | Homozygous | SNP | *IL4* | - |  |
| chr5 | 132007913 | T>A | | Heterozygous | SNP | *IL4* | - |  |
| chr5 | 132011542 | delTGTGTG | | Homozygous | DEL | *IL4* | - |  |
| chr5 | 132011569 | G>A | | Homozygous | SNP | *IL4* | - |  |
| chr5 | 132011575 | A>G | | Homozygous | SNP | *IL4* | - |  |
| chr5 | 132011621 | insTGTG | | Homozygous | INS | *IL4* | - |  |
| chr5 | 132022972 | T>G | | Heterozygous | SNP | *IL4* | - |  |
| chr6 | 31545711 | C>A | | Heterozygous | SNP | *TNF* | - |  |
| chr6 | 31543827 | G>A | | Heterozygous | SNP | *TNF* | - |  |
| chr2 | 113532359 | A>C | | Heterozygous | SNP | *IL1A* | - |  |
| chr2 | 113532885 | G>T | | Heterozygous | SNP | *IL1A* | 0.6779 | 1000 Genomes European |
| chr2 | 113533333 | G>A | | Heterozygous | SNP | *IL1A* | - |  |
| chr2 | 113533339 | A>G | | Heterozygous | SNP | *IL1A* | - |  |
| chr2 | 113534024 | T>C | | Heterozygous | SNP | *IL1A* | - |  |
| chr2 | 113535395 | G>T | | Heterozygous | SNP | *IL1A* | 0.6779 | 1000 Genomes European |
| chr2 | 113535771 | delGT | | Heterozygous | DEL | *IL1A* | 0.4264 | 1000 Genomes European |
| chr2 | 113537352 | T>G | | Heterozygous | SNP | *IL1A* | - |  |
| chr2 | 113537579 | T>C | | Heterozygous | SNP | *IL1A* | 0.6789 | 1000 Genomes European |
| chr2 | 113538490 | A>G | | Homozygous | SNP | *IL1A* | 0.802572 |  |
| chr2 | 113538779 | G>A | | Heterozygous | SNP | *IL1A* | - |  |
| chr2 | 113540205 | G>T | | Heterozygous | SNP | *IL1A* | 0.6779 | 1000 Genomes European |
| chr7 | 22768651 | T>G | | Heterozygous | SNP | *IL6* | - |  |
| chr7 | 22770846 | delT | | Homozygous | DEL | *IL6* | 0.0194 | GnomAD Non-Fin European |
| chr7 | 22767433 | A>G | | Heterozygous | SNP | *IL6* | - |  |
| chr7 | 22768124 | C>A | | Heterozygous | SNP | *IL6* | - |  |
| chr7 | 22768572 | C>G | | Heterozygous | SNP | *IL6* | - |  |
| chr7 | 22768707 | T>G | | Homozygous | SNP | *IL6* | - |  |
| chr3 | 159712441 | T>A | | Heterozygous | SNP | *IL12* | - |  |
| chr3 | 159712993 | G>C | | Homozygous | SNP | *IL12* | - |  |
| chr3 | 159713075 | insGATAGGGTTTAT | | Heterozygous | INS | *IL12* | 0.1501 | 1000 Genomes European |
| chr3 | 159713087 | T>C | | Heterozygous | SNP | *IL12* | - |  |
| chr14 | 24633135 | A>G | | Heterozygous | SNP | *IRF9* | - |  |
| chr14 | 24636433 | C>T | | Heterozygous | SNP | *IRF9* | - |  |
| chr14 | 24638026 | C>T | | Homozygous | SNP | *IRF9* | - |  |
| chr14 | 24638555 | C>T | | Heterozygous | SNP | *IRF9* | - |  |
| **Sample 53** | | | | | | | | |
| **Chr** | **Position** | **Change** | | **Genotype** | **Mutation type** | **Gene** | **Population Freq** | **Group** |
| chr1 | 207498700 | C>T | | Heterozygous | SNP | *CD55* | - |  |
| chr1 | 207510971 | G>T | | Homozygous | SNP | *CD55* | - |  |
| chr1 | 207513719 | del T | | Heterozygous | DEL | *CD55* | 0.0014 | GnomAD Non-Finnish European |
| chr1 | 207489959 | C>T | | Homozygous | SNP | *CD55* | 0.7445 | 1000 Genome European |
| chr1 | 207490035 | A>G | | Heterozygous | SNP | *CD55* | 0.4742 | 1000 Genome European |
| chr1 | 207495427 | G>T | | Heterozygous | SNP | *CD55* | 0.4722 | 1000 Genome European |
| chr1 | 207498644 | C>T | | Heterozygous | SNP | *CD55* | 0.7445 | 1000 Genome European |
| chr1 | 207500293 | C>T | | Homozygous | SNP | *CD55* | 0.7445 | 1000 Genome European |
| chr1 | 207504669 | G>A | | Heterozygous | SNP | *CD55* | 0.7435 | 1000 Genome European |
| chr1 | 207504748 | C>A | | Homozygous | SNP | *CD55* | 0.7435 | 1000 Genome European |
| chr1 | 207504820 | delCA | | Heterozygous | DEL | *CD55* | 0.7376 | 1000 Genome European |
| chr1 | 207510289 | T>C | | Homozygous | SNP | *CD55* | 1 | 1000 Genome European |
| chr1 | 207510548 | A>G | | Heterozygous | SNP | *CD55* | 0.7445 | 1000 Genome European |
| chr1 | 207510596 | G>A | | Homozygous | SNP | *CD55* | 0.7445 | 1000 Genome European |
| chr1 | 207512577 | dupAAT | | Heterozygous | DUP | *CD55* | 1 | 1000 Genome European |
| chr1 | 207532654 | G>A | | Heterozygous | SNP | *CD55* | 1 | 1000 Genome European |
| chr1 | 207532861 | ->T | | Heterozygous | INDEL | *CD55* | 0.2479 | GnomAD Non-Finnish European |
| Chr2 | 113586589 | T>A | | Heterozygous | SNP | *IL1B* | - |  |
| chr2 | 113585147 | T>C | | Heterozygous | SNP | *IL1B* | 0.998 | 1000 Genome European |
| chr2 | 113586469 | A>G | | Homozygous | SNP | *IL1B* | 0.9254 | 1000 Genome European |
| chr2 | 113588553 | A>G | | Heterozygous | SNP | *IL1B* | 0.9264 | 1000 Genome European |
| chr2 | 113588756 | delTT | | Heterozygous | DEL | *IL1B* | 0.2445 | 1000 Genome European |
| chr2 | 113588793 | C>T | | Heterozygous | SNP | *IL1B* | 0.2445 | 1000 Genome European |
| chr2 | 113590390 | G>A | | Homozygous | SNP | *IL1B* | 0.2475 | 1000 Genome European |
| chr2 | 113591275 | A>G | | Heterozygous | SNP | *IL1B* | 0.2485 | 1000 Genome European |
| chr2 | 113591342 | G>A | | Homozygous | SNP | *IL1B* | 0.1769 | 1000 Genome European |
| chr9 | 32468148 | A>G | | Heterozygous | SNP | *DDX58* | - |  |
| chr9 | 32480971 | A>T | | Heterozygous | SNP | *DDX58* | - |  |
| chr9 | 32484889 | A>G | | Heterozygous | SNP | *DDX58* | - |  |
| chr9 | 32489659 | G>A | | Homozygous | SNP | *DDX58* | 0.0021 | GnomAD Non-Finnish European |
| chr9 | 32492207 | G>T | | Heterozygous | SNP | *DDX58* | - |  |
| chr9 | 32493621 | delT | | Homozygous | DEL | *DDX58* | 0.002 | GnomAD Non-Finnish European |
| chr9 | 32499750 | G>A | | Heterozygous | SNP | *DDX58* | 6.5e-5 | GnomAD Non-Finnish European |
| chr9 | 32501180 | A>G | | Heterozygous | SNP | *DDX58* | - |  |
| chr9 | 32504550 | C>A | | Heterozygous | SNP | *DDX58* | - |  |
| chr9 | 32525829 | G>C | | Homozygous | SNP | *DDX58* | - |  |
| chr9 | 32527987 | C>A | | Heterozygous | SNP | *DDX58* | - |  |
| chr9 | 32457558 | A>G | | Homozygous | SNP | *DDX58* | 0.3449 | 1000 Genome European |
| chr9 | 32459450 | T>G | | Heterozygous | SNP | *DDX58* | 0.3529 | 1000 Genome European |
| chr9 | 32466184 | G>A | | Heterozygous | SNP | *DDX58* | 0.4205 | 1000 Genome European |
| chr9 | 32472895 | A>G | | Homozygous | SNP | *DDX58* | 0.3241 | 1000 Genome European |
| chr9 | 32478854 | G>A | | Heterozygous | SNP | *DDX58* | 0.0646 | 1000 Genome European |
| chr9 | 32481501 | delT | | Heterozygous | SNP | *DDX58* | 0.2842 | GnomAD Non-Finnish European |
| chr9 | 32484826 | A>G | | Heterozygous | SNP | *DDX58* | 0.9284 | 1000 Genome European |
| chr9 | 32485288 | dupT | | Homozygous | DUP | *DDX58* | 0.3976 | 1000 Genome European |
| chr9 | 32488237 | G>C | | Heterozygous | SNP | *DDX58* | 0.4205 | 1000 Genome European |
| chr9 | 32488421 | A>G | | Homozygous | SNP | *DDX58* | 0.4195 | 1000 Genome European |
| chr9 | 32490179 | C>T | | Heterozygous | SNP | *DDX58* | 0.4185 | 1000 Genome European |
| chr9 | 32491240 | C>T | | Heterozygous | SNP | *DDX58* | 0.5577 | 1000 Genome European |
| chr9 | 32504474 | G>A | | Heterozygous | SNP | *DDX58* | 0.2068 | 1000 Genome European |
| chr9 | 32525915 | C>G | | Homozygous | SNP | *DDX58* | 0.2078 | 1000 Genome European |
| chr9 | 32525969 | C>A | | Heterozygous | SNP | *DDX58* | 0.6243 | 1000 Genome European |
| chr9 | 32526233 | G>C | | Homozygous | SNP | *DDX58* | 0.6262 | 1000 Genome European |
| chr21 | 42846401 | G>A | | Heterozygous | SNP | *TMPRSS2* | - |  |
| chr9 | 42847135 | A>G | | Heterozygous | SNP | *TMPRSS2* | - |  |
| chr21 | 42847573 | A>G | | Homozygous | SNP | *TMPRSS2* | 0 | GnomAD Non-Finnish European |
| chr21 | 42857016 | T>C | | Heterozygous | SNP | *TMPRSS2* | - |  |
| chr21 | 42857088 | A>G | | Heterozygous | SNP | *TMPRSS2* | - |  |
| chr21 | 42840580 | T>C | | Heterozygous | SNP | *TMPRSS2* | 0.7843 | 1000 Genome European |
| chr21 | 42840595 | G>C | | Homozygous | SNP | *TMPRSS2* | 0.7843 | 1000 Genome European |
| chr21 | 42840872 | T>C | | Heterozygous | SNP | *TMPRSS2* | 0.9811 | 1000 Genome European |
| chr21 | 42847036 | A>G | | Heterozygous | SNP | *TMPRSS2* | 0.9801 | 1000 Genome European |
| chr21 | 42847185 | G>C | | Heterozygous | SNP | *TMPRSS2* | 0.3943 | GnomAD Non-Finnish European |
| chr21 | 42847606 | G>C | | Homozygous | SNP | *TMPRSS2* | 0.4571 | GnomAD Non-Finnish European |
| chr21 | 42856949 | T>C | | Heterozygous | SNP | *TMPRSS2* | 0.9781 | 1000 Genome European |
| chr21 | 42859067 | G>C | | Heterozygous | SNP | *TMPRSS2* | 0.7147 | 1000 Genome European |
| chr21 | 42859222 | G>C | | Heterozygous | SNP | *TMPRSS2* | 0.7147 | 1000 Genome European |
| chr21 | 42859876 | C>T | | Homozygous | SNP | *TMPRSS2* | 0.7127 | 1000 Genome European |
| chr21 | 42860485 | G>C | | Heterozygous | SNP | *TMPRSS2* | 0.6998 | 1000 Genome European |
| chrX | 15609609 | G>T | | Homozygous | SNP | *ACE 2* | - |  |
| chr11 | 612394 | T>C | | Heterozygous | SNP | *IRF 7* | - |  |
| chr11 | 616375 | A>G | | Heterozygous | SNP | *IRF 7* | - |  |
| chr11 | 612843 | T>G | | Homozygous | SNP | *IRF 7* | 0.2674 | 1000 Genome European |
| chr11 | 612967 | A>G | | Heterozygous | SNP | *IRF 7* | 0.2674 | 1000 Genome European |
| chr11 | 613165 | G>C | | Homozygous | SNP | *IRF 7* | 0.2674 | 1000 Genome European |
| chr11 | 613192 | A>G | | Heterozygous | SNP | *IRF 7* | 0.2674 | 1000 Genome European |
| chr11 | 613208 | T>C | | Heterozygous | SNP | *IRF 7* | 0.2674 | 1000 Genome European |
| chr11 | 613297 | T>G | | Heterozygous | SNP | *IRF 7* | 0.2793 | 1000 Genome European |
| chr11 | 613643 | delTGTGAGTGACGGGGGTGGGCGGGGACAGGA | | Homozygous | DEL | *IRF 7* | 0.0175 | GnomAD Non-Finnish European |
| chr11 | 613697 | A>G | | Heterozygous | SNP | *IRF 7* | 0.2058 | GnomAD Non-Finnish European |
| chr11 | 613742 | G>A | | Heterozygous | SNP | *IRF 7* | 0.2684 | 1000 Genome European |
| chr11 | 613757 | A>G | | Homozygous | SNP | *IRF 7* | 0.2694 | 1000 Genome European |
| chr11 | 614318 | T>C | | Heterozygous | SNP | *IRF 7* | 0.2674 | 1000 Genome European |
| chr11 | 614864 | C>T | | Heterozygous | SNP | *IRF 7* | 0.8748 | 1000 Genome European |
| chr11 | 615010 | G>T | | Heterozygous | SNP | *IRF 7* | 0.2674 | 1000 Genome European |
| chr11 | 615011 | A>T | | Homozygous | SNP | *IRF 7* | 0.2674 | 1000 Genome European |
| chr5 | 132006844 | G>C | | Heterozygous | SNP | *IL4* | 0.9672 | 1000 Genome European |
| chr5 | 132009523 | T>A | | Homozygous | SNP | *IL4* | - |  |
| chr5 | 132009710 | C>T | | Heterozygous | SNP | *IL4* | 0.168 | 1000 Genome European |
| chr5 | 132018132 | A>G | | Heterozygous | SNP | *IL4* | 0.168 | 1000 Genome European |
| chr5 | 132018169 | C>A | | Heterozygous | SNP | *IL4* | 0.17 | 1000 Genome European |
| chr7 | 22767433 | A>G | | Heterozygous | SNP | *IL6* | 0.5895 | 1000 Genome European |
| chr7 | 22768124 | C>A | | Homozygous | SNP | *IL6* | 0.5885 | 1000 Genome European |
| chr7 | 22769103 | A>G | | Heterozygous | SNP | *IL6* | - |  |
| chr7 | 22770846 | delAA | | Heterozygous | SNP | *IL6* | 0.0008 | GnomAD Non-Finnish European |
| chr11 | 24631687 | A>G | | Heterozygous | SNP | *IRF9* | - |  |
| chr11 | 24631774 | G>T | | Homozygous | SNP | *IRF9* | - |  |
| chr11 | 24633114 | G>T | | Heterozygous | SNP | *IRF9* | - |  |
| chr11 | 24638026 | C>T | | Heterozygous | SNP | *IRF9* | 0.2624 | 1000 Genome European |
| chr11 | 24639038 | A>T | | Heterozygous | SNP | *IRF9* | - |  |
| chr11 | 123373133 | T>C | | Homozygous | SNP | *IL2* | 0.2962 | 1000 Genome European |
| chr11 | 123374632 | G>A | | Heterozygous | SNP | *IL2* | - |  |
| chr2 | 123374820 | delT | | Heterozygous | del | *IL2* | 0.175 | GnomAD Non-Finnish European |
| chr2 | 123377482 | C>A | | Homozygous | SNP | *IL2* | 0.3449 | 1000 Genome European |
| chr2 | 123377980 | A>C | | Homozygous | SNP | *IL2* | 0.2922 | 1000 Genome European |

**Supplementary Table 3.** Age-, sex-, and comorbidity-stratified analysis to reveal the effect of these factors to the differences between the patients within the symptom groups.

| **rs number** | **Position** | **Change** | **Mild symptom group** | | **Moderate symptom group** | | **Severe symptom group** | |
| --- | --- | --- | --- | --- | --- | --- | --- | --- |
| ***CD55*** | | | | | | | | |
| rs11120753 | 1:207527285 | G>A | Age group 1 (%) | Age group 2 (%) | Age group 1 (%) | Age group 2 (%) | Age group 1 (%) | Age group 2 (%) |
| N = 7 | | | 50 | 50 | 0 | 100 | 0 | 100 |
| p-value | | | p = 1 | | **p = 1.58x10^-30^** | | **p = 1.58x10^-30^** | |
|  | | | Female (%) | Male (%) | Female (%) | Male (%) | Female (%) | Male (%) |
|  | | | 25 | 75 | 100 | 0 | 0 | 100 |
| p-value | | | **p = 5.64x10^-7^** | | **p = 1.58x10^-30^** | | **p = 1.58x10^-30^** | |
|  | | | Reported comorbidities (%) | No reported comorbidities (%) | Reported comorbidities (%) | No reported comorbidities (%) | Reported comorbidities (%) | No reported comorbidities (%) |
|  | | | 50 | 50 | 50 | 50 | 100 | 0 |
| p-value | | | p = 1 | | p = 1 | | **p = 1.58x10^-30^** | |
| ***IL1B*** | | | | | | | | |
| rs1681980552 | 2:113588756 | delAAA | Age group 1 (%) | Age group 2 (%) | Age group 1 (%) | Age group 2 (%) | Age group 1 (%) | Age group 2 (%) |
| N = 10 | | | 33 | 67 | 67 | 33 | 50 | 50 |
| p-value | | | **p = 0.000874** | | **p = 0.000874** | | p = 1 | |
|  | | | Female (%) | Male (%) | Female (%) | Male (%) | Female (%) | Male (%) |
|  | | | 33 | 67 | 33 | 67 | 50 | 50 |
| p-value | | | **p = 0.000874** | | **p = 0.000874** | | p = 1 | |
|  | | | Reported comorbidities (%) | No reported comorbidities (%) | Reported comorbidities (%) | No reported comorbidities (%) | Reported comorbidities (%) | No reported comorbidities (%) |
|  | | | 67 | 33 | 67 | 33 | 75 | 25 |
| p-value | | | **p = 0.000874** | | **p = 0.000874** | | **p = 5.64x10^-7^** | |
| rs1143634 | 2:113590390 | G>A | Age group 1 (%) | Age group 2 (%) | Age group 1 (%) | Age group 2 (%) | Age group 1 (%) | Age group 2 (%) |
| N = 8 | | | 100 | 0 | 100 | 0 | 0 | 100 |
| p-value | | | **p = 1.58x10^-30^** | | **p = 1.58x10^-30^** | | **p = 1.58x10^-30^** | |
|  | | | Female (%) | Male (%) | Female (%) | Male (%) | Female (%) | Male (%) |
|  | | | 0 | 100 | 0 | 100 | 40 | 60 |
| p-value | | | **p = 1.58x10^-30^** | | **p = 1.58x10^-30^** | | p = 0.057 | |
|  | | | Reported comorbidities (%) | No reported comorbidities (%) | Reported comorbidities (%) | No reported comorbidities (%) | Reported comorbidities (%) | No reported comorbidities (%) |
|  | | | 0 | 100 | 0 | 100 | 100 | 0 |
| p-value | | | **p = 1.58x10^-30^** | | **p = 1.58x10^-30^** | | **p = 1.58x10^-30^** | |
| ***IL4*** | | | | | | | | |
| rs2243290 | 5:132018169 | C>A | Age group 1 (%) | Age group 2 (%) | Age group 1 (%) | Age group 2 (%) | Age group 1 (%) | Age group 2 (%) |
| N = 14 | | | 0 | 100 | 20 | 80 | 17 | 83 |
| p-value | | | **p = 1.58x10^-30^** | | **p = 1.12x10^-9^** | | **p = 1.31x10^-11^** | |
|  | | | Female (%) | Male (%) | Female (%) | Male (%) | Female (%) | Male (%) |
|  | | | 100 | 0 | 40 | 60 | 50 | 50 |
| p-value | | | **p = 1.58x10^-30^** | | p = 0.057 | | p = 1 | |
|  | | | Reported comorbidities (%) | No reported comorbidities (%) | Reported comorbidities (%) | No reported comorbidities (%) | Reported comorbidities (%) | No reported comorbidities (%) |
|  | | | 100 | 0 | 100 | 0 | 83 | 17 |
| p-value | | | **p = 1.58x10^-30^** | | **p = 1.58x10^-30^** | | **p = 1.31x10^-11^** | |
| ***IRF7*** | | | | | | | | |
| rs34948036 | 11:612823 | insT | Age group 1 (%) | Age group 2 (%) | Age group 1 (%) | Age group 2 (%) | Age group 1 (%) | Age group 2 (%) |
| N = 15 | | | 75 | 25 | 25 | 75 | 29 | 71 |
| p-value | | | **p = 5.64x10^-7^** | | **p = 5.64x10^-7^** | | **p = 0.000032** | |
|  | | | Female (%) | Male (%) | Female (%) | Male (%) | Female (%) | Male (%) |
|  | | | 25 | 75 | 50 | 50 | 29 | 71 |
| p-value | | | **p = 5.64x10^-7^** | | p = 1 | | **p = 0.000032** | |
|  | | | Reported comorbidities (%) | No reported comorbidities (%) | Reported comorbidities (%) | No reported comorbidities (%) | Reported comorbidities (%) | No reported comorbidities (%) |
|  | | | 50 | 50 | 25 | 75 | 86 | 14 |
| p-value | | | p = 1 | | **p = 5.64x10^-7^** | | **p = 8.28x10^-14^** | |
| rs1051390 | 11:613165 | G>C | Age group 1 (%) | Age group 2 (%) | Age group 1 (%) | Age group 2 (%) | Age group 1 (%) | Age group 2 (%) |
| N = 8 | | | 50 | 50 | 0 | 100 | 0 | 100 |
| p-value | | | p = 1 | | **p = 1.58x10^-30^** | | **p = 1.58x10^-30^** | |
|  | | | Female (%) | Male (%) | Female (%) | Male (%) | Female (%) | Male (%) |
|  | | | 50 | 50 | 100 | 0 | 40 | 60 |
| p-value | | | p = 1 | | **p = 1.58x10^-30^** | | p = 0.057 | |
|  | | | Reported comorbidities (%) | No reported comorbidities (%) | Reported comorbidities (%) | No reported comorbidities (%) | Reported comorbidities (%) | No reported comorbidities (%) |
|  | | | 50 | 50 | 100 | 0 | 100 | 0 |
| p-value | | | p = 1 | | **p = 1.58x10^-30^** | | **p = 1.58x10^-30^** | |
| rs12422022 | 11:613192 | A>G | Age group 1 (%) | Age group 2 (%) | Age group 1 (%) | Age group 2 (%) | Age group 1 (%) | Age group 2 (%) |
| N = 8 | | | 50 | 50 | 0 | 100 | 0 | 100 |
| p-value | | | p = 1 | | **p = 1.58x10^-30^** | | **p = 1.58x10^-30^** | |
|  | | | Female (%) | Male (%) | Female (%) | Male (%) | Female (%) | Male (%) |
|  | | | 50 | 50 | 100 | 0 | 40 | 60 |
| p-value | | | p = 1 | | **p = 1.58x10^-30^** | | p = 0.057 | |
|  | | | Reported comorbidities (%) | No reported comorbidities (%) | Reported comorbidities (%) | No reported comorbidities (%) | Reported comorbidities (%) | No reported comorbidities (%) |
|  | | | 50 | 50 | 100 | 0 | 100 | 0 |
| p-value | | | p = 1 | | **p = 1.58x10^-30^** | | **p = 1.58x10^-30^** | |
| rs1131665 | 11:613208 | T>C | Age group 1 (%) | Age group 2 (%) | Age group 1 (%) | Age group 2 (%) | Age group 1 (%) | Age group 2 (%) |
| N = 8 | | | 50 | 50 | 0 | 100 | 0 | 100 |
| p-value | | | p = 1 | | **p = 1.58x10^-30^** | | **p = 1.58x10^-30^** | |
|  | | | Female (%) | Male (%) | Female (%) | Male (%) | Female (%) | Male (%) |
|  | | | 50 | 50 | 100 | 0 | 40 | 60 |
| p-value | | | p = 1 | | **p = 1.58x10^-30^** | | p = 0.057 | |
|  | | | Reported comorbidities (%) | No reported comorbidities (%) | Reported comorbidities (%) | No reported comorbidities (%) | Reported comorbidities (%) | No reported comorbidities (%) |
|  | | | 50 | 50 | 100 | 0 | 100 | 0 |
| p-value | | | p = 1 | | **p = 1.58x10^-30^** | | **p = 1.58x10^-30^** | |
| ***DDX58*** | | | | | | | | |
| rs10813831 | 9:32526146 | G>A | Age group 1 (%) | Age group 2 (%) | Age group 1 (%) | Age group 2 (%) | Age group 1 (%) | Age group 2 (%) |
| N = 7 | | | 100 | 0 | 0 | 100 | 0 | 100 |
| p-value | | | **p = 1.58x10^-30^** | | **p = 1.58x10^-30^** | | **p = 1.58x10^-30^** | |
|  | | | Female (%) | Male (%) | Female (%) | Male (%) | Female (%) | Male (%) |
|  | | | 50 | 50 | 100 | 0 | 0 | 100 |
| p-value | | | p = 1 | | **p = 1.58x10^-30^** | | **p = 1.58x10^-30^** | |
|  | | | Reported comorbidities (%) | No reported comorbidities (%) | Reported comorbidities (%) | No reported comorbidities (%) | Reported comorbidities (%) | No reported comorbidities (%) |
|  | | | 50 | 50 | 100 | 0 | 100 | 0 |
| p-value | | | p = 1 | | **p = 1.58x10^-30^** | | **p = 1.58x10^-30^** | |
| rs1213032873 | 9:32485288 | insA | Age group 1 (%) | Age group 2 (%) | Age group 1 (%) | Age group 2 (%) | Age group 1 (%) | Age group 2 (%) |
| N = 2 | | | 0 | 0 | 0 | 0 | 100 | 0 |
| p-value | | | n/a | | n/a | | **p = 1.58x10^-30^** | |
|  | | | Female (%) | Male (%) | Female (%) | Male (%) | Female (%) | Male (%) |
|  | | | 0 | 0 | 0 | 0 | 50 | 50 |
| p-value | | | n/a | | n/a | | p = 1 | |
|  | | | Reported comorbidities (%) | No reported comorbidities (%) | Reported comorbidities (%) | No reported comorbidities (%) | Reported comorbidities (%) | No reported comorbidities (%) |
|  | | | 0 | 0 | 0 | 0 | 100 | 0 |
| p-value | | | n/a | | n/a | | **p = 1.58x10^-30^** | |
| ***TMPRSS2*** | | | | | | | | |
| rs17854725 | 21:42845383 | A>G | Age group 1 (%) | Age group 2 (%) | Age group 1 (%) | Age group 2 (%) | Age group 1 (%) | Age group 2 (%) |
| N = 24 | | | 75 | 25 | 25 | 75 | 17 | 83 |
| p-value | | | **p = 5.64x10^-7^** | | **p = 5.64x10^-7^** | | **p = 1.31x10^-11^** | |
|  | | | Female (%) | Male (%) | Female (%) | Male (%) | Female (%) | Male (%) |
|  | | | 50 | 50 | 50 | 50 | 25 | 75 |
| p-value | | | p = 1 | | p = 1 | | **p = 5.64x10^-7^** | |
|  | | | Reported comorbidities (%) | No reported comorbidities (%) | Reported comorbidities (%) | No reported comorbidities (%) | Reported comorbidities (%) | No reported comorbidities (%) |
|  | | | 38 | 62 | 25 | 75 | 83 | 17 |
| p-value | | | **p = 0.021** | | **p = 5.64x10^-7^** | | **p = 1.31x10^-11^** | |
| rs73230068 | 21:42845167 | G>C | Age group 1 (%) | Age group 2 (%) | Age group 1 (%) | Age group 2 (%) | Age group 1 (%) | Age group 2 (%) |
| N = 4 | | | 0 | 0 | 0 | 100 | 50 | 50 |
| p-value | | | n/a | | **p = 1.58x10^-30^** | | p = 1 | |
|  | | | Female (%) | Male (%) | Female (%) | Male (%) | Female (%) | Male (%) |
|  | | | 0 | 0 | 25 | 75 | 0 | 100 |
| p-value | | | n/a | | **p = 5.64x10^-7^** | | **p = 1.58x10^-30^** | |
|  | | | Reported comorbidities (%) | No reported comorbidities (%) | Reported comorbidities (%) | No reported comorbidities (%) | Reported comorbidities (%) | No reported comorbidities (%) |
|  | | | 0 | 0 | 50 | 50 | 50 | 50 |
| p-value | | | n/a | | p = 1 | | p = 1 | |
| ***ACE2*** | | | | | | | | |
| rs2285666 | X:15610348 | C>T | Age group 1 (%) | Age group 2 (%) | Age group 1 (%) | Age group 2 (%) | Age group 1 (%) | Age group 2 (%) |
| N = 9 | | | 33 | 67 | 40 | 60 | 0 | 100 |
| p-value | | | **p = 0.000874** | | p = 0.057 | | **p = 1.58x10^-30^** | |
|  | | | Female (%) | Male (%) | Female (%) | Male (%) | Female (%) | Male (%) |
|  | | | 100 | 0 | 60 | 40 | 0 | 100 |
| p-value | | | **p = 1.58x10^-30^** | | p = 0.057 | | **p = 1.58x10^-30^** | |
|  | | | Reported comorbidities (%) | No reported comorbidities (%) | Reported comorbidities (%) | No reported comorbidities (%) | Reported comorbidities (%) | No reported comorbidities (%) |
|  | | | 67 | 33 | 20 | 80 | 100 | 0 |
| p-value | | | **p = 0.000874** | | **p = 1.12x10^-9^** | | **p = 1.58x10^-30^** | |
